# Photon Versus Proton Whole-Body Irradiation Combined with Olaparib in C57BL/6 Mice: An Exploratory Study of Oxidative Stress, Inflammatory, and DNA Damage Response Biomarkers

**DOI:** 10.3390/antiox15101365

**Published:** 2026-10-21

**Authors:** Dinu Stefan, Justine Lequesne, Frédéric Pouzoulet, Ludovic De Marzi, Frédérique Mégnin-Chanet, François Christy, Cyril Moignier, Bénédicte Clarisse, Victor Barraux, Mathilde Lechevrel, Stéphanie Gente, François Sichel, Carine Laurent

**Affiliations:** 1 Radiation Oncology, Centre François Baclesse, 3 Avenue du Général Harris, 14000 Caen, France; 2 ABTE-EA4651, Normandie University, UNICAEN, 14000 Caen Cedex, France; v.barraux@baclesse.unicancer.fr (V.B.); carine.laurent@unicaen.fr (C.L.); 3 CYCLHAD, 14000 Caen, France; 4 Clinical Research, Centre François Baclesse, 14000 Caen, France; 5 IRIS, UMR9029/U1353, 91400 Orsay, France; 6 Proton Therapy Center, Institut Curie, 91400 Orsay, France; 7 INSERM U1196/UMR9187, Université Paris Saclay, 91405 Orsay, France; 8 Medical Physics, Centre François Baclesse, 14000 Caen, France; 9 Sem Saphyn, Archade, 14000 Caen, France

**Keywords:** olaparib, PARP inhibitor, proton therapy, photon irradiation, oxidative stress, antioxidant enzymes, glutathione redox status, superoxide dismutase, whole-body irradiation, acute radiation syndrome, reactive oxygen species

## Abstract

Olaparib, a poly(ADP-ribose) polymerase (PARP) inhibitor with established radiosensitizing properties, is a candidate for combination with radiotherapy, yet its multi-organ oxidative toxicity when combined with photon versus proton irradiation has not been characterized. In the ToxIP3 project, C57BL/6 male mice were allocated to six groups (n = 10 per group) receiving whole-body irradiation at 7.5 Gy by photons (TrueBeam™) or protons (190.6 MeV, Institut Curie, Orsay), with or without concurrent oral olaparib (50 mg/kg/day). Seventy-four biomarkers spanning protein carbonylation, lipid peroxidation, antioxidant enzyme activities, glutathione redox status, PARP activity, total protein, TGF-β1, and 13 plasma cytokines were quantified across nine compartments. The principal comparison (protons + olaparib vs. photons + olaparib) used two-sided Wilcoxon–Mann–Whitney tests with Benjamini–Hochberg false discovery rate (FDR) correction; time-shifted comparisons used rank-based ANCOVA adjusted for the day of sacrifice. Combination arms showed markedly increased lethality (median survival 9 days for photons + olaparib and 8 days for protons + olaparib in all 60 randomized animals); 54 mice were available for biomarker analysis. Of 74 biomarkers, 18 differed between combination arms after FDR correction; seven hepatic protein-normalized measurements were considered non-interpretable, leaving 11. Protons + olaparib was associated with higher intestinal GPx and GSH/GSSG, higher erythrocyte GSH, GSSG and SOD, higher cerebral SOD (+75.1%, q = 0.010), and a higher hepatic GSH/GSSG ratio (+108.2%, q < 0.001), whereas skin showed a lower GSH/GSSG ratio (−77.6%) and higher protein carbonylation (+142.9%). These exploratory findings, obtained in a near-lethal whole-body irradiation model, show modality-associated differences in redox biomarkers but do not establish differences in normal-tissue toxicity.

## 1. Introduction

Olaparib (Lynparza, AstraZeneca) was the first PARP-1/2 inhibitor to reach regulatory approval in platinum-sensitive relapsed ovarian cancer, receiving conditional EMA authorization in 2014 based on the Phase II Study 19 trial in BRCA-mutated patients, with the indication broadened by the FDA in 2017 and later established as first-line maintenance therapy following the pivotal SOLO1 trial [1,2]. As a competitive inhibitor of the NAD+ catalytic site, olaparib interferes with single-strand break (SSB) repair via the base excision repair (BER) pathway [3] and induces synthetic lethality in tumours harbouring homologous recombination (HR) deficiencies [4,5,6]. Beyond catalytic inhibition, olaparib traps PARP-1 on DNA, generating cytotoxic protein–DNA adducts that collapse replication forks and produce double-strand breaks (DSBs) requiring HR for resolution [7].

The combination of PARPi with radiotherapy represents a clinically attractive strategy, since ionizing radiation induces precisely the SSBs whose repair depends on the pathways targeted by these agents [8]. Radiosensitizing properties of olaparib have been extensively documented preclinically [9,10], and clinical phase I trials have confirmed feasibility, albeit with significant dose-limiting normal-tissue toxicity, including hematological, gastrointestinal, and cutaneous late effects [11,12,13]. Extended follow-up has identified severe late toxicity in some long-term survivors [12].

Photons and higher linear energy transfer (LET) irradiations (protons, carbon ions) differ fundamentally in their physical and biological properties. Photons (LET ≈ 0.2–2 keV/µm) generate sparsely ionizing tracks producing predominantly isolated SSBs and simple DSBs, repaired principally by NHEJ. In clinical proton therapy, the spread-out Bragg peak (SOBP) is shaped to conform to the tumour volume, where LET rises sharply to ~7–10 keV/µm, providing markedly increased biological effectiveness at the target itself through distinct track-structure characteristics that generate a higher proportion of complex, spatially clustered DSBs requiring HR for faithful resolution. By contrast, the entrance plateau (LET ~0.5–1 keV/µm) upstream of the SOBP irradiates the surrounding healthy tissue at a sparse ionization pattern comparable to that of megavoltage photons [14,15,16,17,18]. Since olaparib impairs HR initiation by sequestering PARP-1 on chromatin [7], its interaction with high-LET-induced damage is mechanistically distinct. Emerging data further suggest a stronger PARPi radiosensitizing effect at higher linear energy transfer, encouraging the use of this combination to improve tumour control in clinical situations where particle therapy is indicated [19,20,21]. Whether olaparib’s interaction with proton-induced damage translates into differential normal-tissue toxicity independently of this high-LET radiosensitizing effect remains unknown.

Beyond these physical differences, the biological response to PARP inhibition also varies across tumours and normal tissues. PARP-1 expression is generally higher in tumours, correlating with aggressiveness [22,23], and comparatively low in normal tissues—particularly the brain—but higher in proliferative and hematopoietic compartments [23,24]. This heterogeneity is mirrored clinically. Olaparib was safely combined with brain irradiation at comparatively higher doses without dose-limiting neurotoxicity [13], whereas tolerated doses were lower in head-and-neck [25] and breast irradiation [12]. As this landscape has been established almost exclusively from separate, single-organ photon trials, we used whole-body irradiation to compare tissue-specific toxicity systematically within the same animals. Our configuration deliberately departs from clinical proton radiotherapy, where normal-tissue exposure is localized, spatially heterogeneous, and generally fractionated, and instead produces systemic acute radiation syndrome. It also allows the maximum number of organs to be harvested per animal, as required by the approved protocol and in accordance with the reduction principle. The model is accordingly intended as a screen, its purpose being to identify the compartments in which modality-dependent differences are largest, so that these can subsequently be re-examined in localized, fractionated, tumour-bearing models.

The single whole-body dose was determined in a prior dose-titration experiment evaluating survival curves at 0, 6, 7.5, 10, 12, and 14 Gy, which established 7.5 Gy as the highest dose compatible with prolonged survival after whole-body photon or proton irradiation alone. It was also the dose at which oxidative stress, inflammation, and genotoxicity biomarkers were quantified in that study and shown to be measurably altered in the compartments examined there [26]. Although post-exposure PARP inhibition following low-dose total-body irradiation has been reported to attenuate hematopoietic and oxidative injury in mice [27], the comparative effects of photon and proton irradiation in the presence of olaparib have not been investigated across multiple compartments within the same animals. No combination-specific dose-finding step was performed. The 7.5 Gy dose was therefore retained to maximize the ability to detect and characterize potential differences between photon and proton irradiation, which constituted the primary objective of this exploratory study. Because modality-dependent differences were expected to be smaller than the overall effect of irradiation, reducing the radiation dose could have compromised the ability to detect such differences within a design comprising ten animals per group. Although a staged, combination-specific dose-escalation approach might have identified a lower effective dose, it would not necessarily have ensured detectable responses across all compartments and would have required substantial additional animal use for an uncertain outcome. 

Radiation-induced oxidative stress is a primary mechanism of normal-tissue damage. Reactive oxygen species (ROS) generated by water radiolysis propagate oxidative chain reactions targeting DNA, proteins (carbonylation), and lipid membranes (lipid peroxidation/MDA), while enzymatic antioxidant systems—SOD, catalase, GPx—and the non-enzymatic GSH/GSSG redox buffer constitute the first-line defence. PARP-1 also regulates NAD+ availability and NFκB-mediated antioxidant gene expression [28], such that its inhibition may modulate tissue antioxidant capacity in a radiation modality-dependent manner.

To our knowledge, no study has systematically compared multi-organ redox biomarkers of olaparib combined with whole-body photon versus proton irradiation. The present exploratory study addresses this gap by quantifying 74 biomarkers of oxidative stress, DNA damage, antioxidant defences, and inflammation across nine biological compartments of C57BL/6 mice exposed to whole-body irradiation, by photons or by protons, with or without olaparib.

We hypothesized that, within this whole-body screening model, combining olaparib with proton rather than photon irradiation would be associated with different tissue redox-biomarker profiles, and we sought to identify the compartments in which such modality-associated differences were largest.

## 2. Materials and Methods

### 2.1. Animal Model and Experimental Design

Sixty male C57BL/6 mice (11 weeks, 20–30 g) were randomly allocated to six groups: control, olaparib alone, photon irradiation, proton irradiation, photons + olaparib, and protons + olaparib. Group size (n = 10 per arm) was determined a priori using G*Power version 3.1.9.7 software, based on detecting a biologically meaningful 25% between-group difference by a two-sided *t*-test (α = 0.05). The C57BL/6 strain was chosen as the reference immunocompetent background for radiation normal-tissue toxicity research: it is the most extensively characterized strain for radiation-induced oxidative stress and antioxidant responses, offers well-defined whole-body irradiation dose–response data, and ensures continuity with our previously validated model [26]. Compared with more radiosensitive strains (e.g., BALB/c) or outbred stocks, C57BL/6 provides an intermediate, reproducible radiation-response phenotype with low genetic variability, favouring detection of modality- and drug-dependent effects [29]. A male-only cohort was used to limit potential variability associated with sex-hormone and oestrous-cycle-dependent modulation of oxidative stress and glutathione metabolism [30]; this design precludes evaluation of sex as a biological variable, and the findings cannot be extrapolated to females. Animals were housed under standard conditions (12 h light/dark cycle, 22 ± 2 °C, ad libitum food and water). All procedures complied with EU Directive 2010/63/EU and were approved under project authorization APAFIS#26093-202006172228866 v1, granted by the French Ministry of Higher Education, Research and Innovation (18 June 2020) following a favourable opinion from Ethics Committee n°118. The combined olaparib–irradiation regimen was assigned a severity classification of “severe” (“Classe sévère”). Upon completion, the project will undergo a mandatory retrospective ethical review.

Irradiation and drug administration

Whole-body irradiation (WBI) was delivered as a single 7.5 Gy fraction, prescribed as a physical dose. No relative biological effectiveness (RBE) correction was applied to the proton arm so that both modalities were compared at an identical physical dose. The generic clinical proton RBE of 1.1 is intended to account for the elevated linear energy transfer (LET) across the spread-out Bragg peak, and particularly at its distal edge, where in vivo studies report RBE increases from ~1.1 in the entrance region to 1.3–1.35 at the distal edge [31,32]. Here, however, irradiation was delivered in the entrance plateau, where the LET (~0.5–1 keV/µm) and the corresponding RBE approach those of megavoltage photons [17,18]. Matching the two arms at an equal physical dose keeps them comparable in the absorbed dose; residual differences in beam quality, dose rate, and delivery technique cannot be excluded as contributors to the observed differences. The 7.5 Gy dose was derived from a prior dose-titration study conducted by our group in the same strain, facility, and beam configuration, in which 7.5 Gy was the highest dose compatible with prolonged survival. For this exploratory screen of 74 biomarkers across nine compartments, this maximal tolerated dose was chosen deliberately—rather than a lower, conventionally fractionated dose—to maximize effect size and thus the sensitivity for detecting radiation-induced changes in redox balance, inflammation, and PARP activity across all organs, including late-responding, low-α/β tissues such as brain and liver [26].

Both photon and proton irradiations were performed at the Institut Curie Proton Therapy Centre (Orsay) on the CurieCoreTech Experimental Radiotherapy platform (RadeXp). Photon WBI was delivered on a TrueBeam™ (Varian Medical Systems, Palo Alto, CA, USA) linac (600 MU/min, 10 mm bolus) at a dose rate of approximately 6 Gy/min at the prescription depth (≈75 s for 7.5 Gy). Proton WBI was delivered on a C230 IBA cyclotron (IBA, Ion Beam Applications, Louvain-la-Neuve, Belgium) using pencil-beam scanning (PBS) in the entrance plateau of the Bragg peak (190.6 MeV; LET ≈ 0.5–1 keV/µm; ≈32 Gy/min; ≈14 s for 7.5 Gy), isolating proton track-structure effects from high-LET Bragg peak biology [17,33]. Dose rates are reported as mean values (delivered dose divided by total delivery time). Although the two delivery techniques differ intrinsically in dose rate, both remain well within the conventional range and far below the ultra-high-dose-rate (FLASH) threshold (>40 Gy/s ≈ 2400 Gy/min); no dose-rate-dependent modulation of oxidative or inflammatory responses is therefore expected to confound the modality comparison.

The 190.6 MeV energy was selected as the highest available energy so as to obtain a dose as homogeneous as possible in the entrance-plateau region. In PBS mode, a 5 × 5 cm^2^ field was generated by scanning a single, narrow, monoenergetic beam on the central axis (spot size ≈4.5 mm at isocentre; uniform, equally weighted grid; 3 mm centre-to-centre spacing). The absolute dose per monitor unit was determined at field centre, in the plateau of the pseudo-monoenergetic 190.6 MeV pristine Bragg peak (3 g/cm^2^ depth), following IAEA TRS-398, using a plane-parallel ionization chamber (PPC05, IBA Dosimetry, Belgium) cross-calibrated for absorbed dose to water against a Semiflex chamber traceable to a ^60^Co beam at the French national metrology institute (CEA-LNHB). Delivered doses were independently verified by thermoluminescent dosimetry (within ±5% of prescription; homogeneity ±3%) [34,35]. The estimated absorbed dose to each studied compartment is reported for both modalities in Supplementary Table S1, both in absolute terms and as a percentage of the prescribed whole-body dose. The comparable compartmental doses achieved with the two modalities (97–104% of the prescribed dose; SD ≤ 0.05 Gy) indicate that the divergent biomarker responses between arms are unlikely to be explained solely by differences in absorbed dose, supporting a potential contribution of modality-specific radiation quality and track-structure effects.

**Figure 1:**
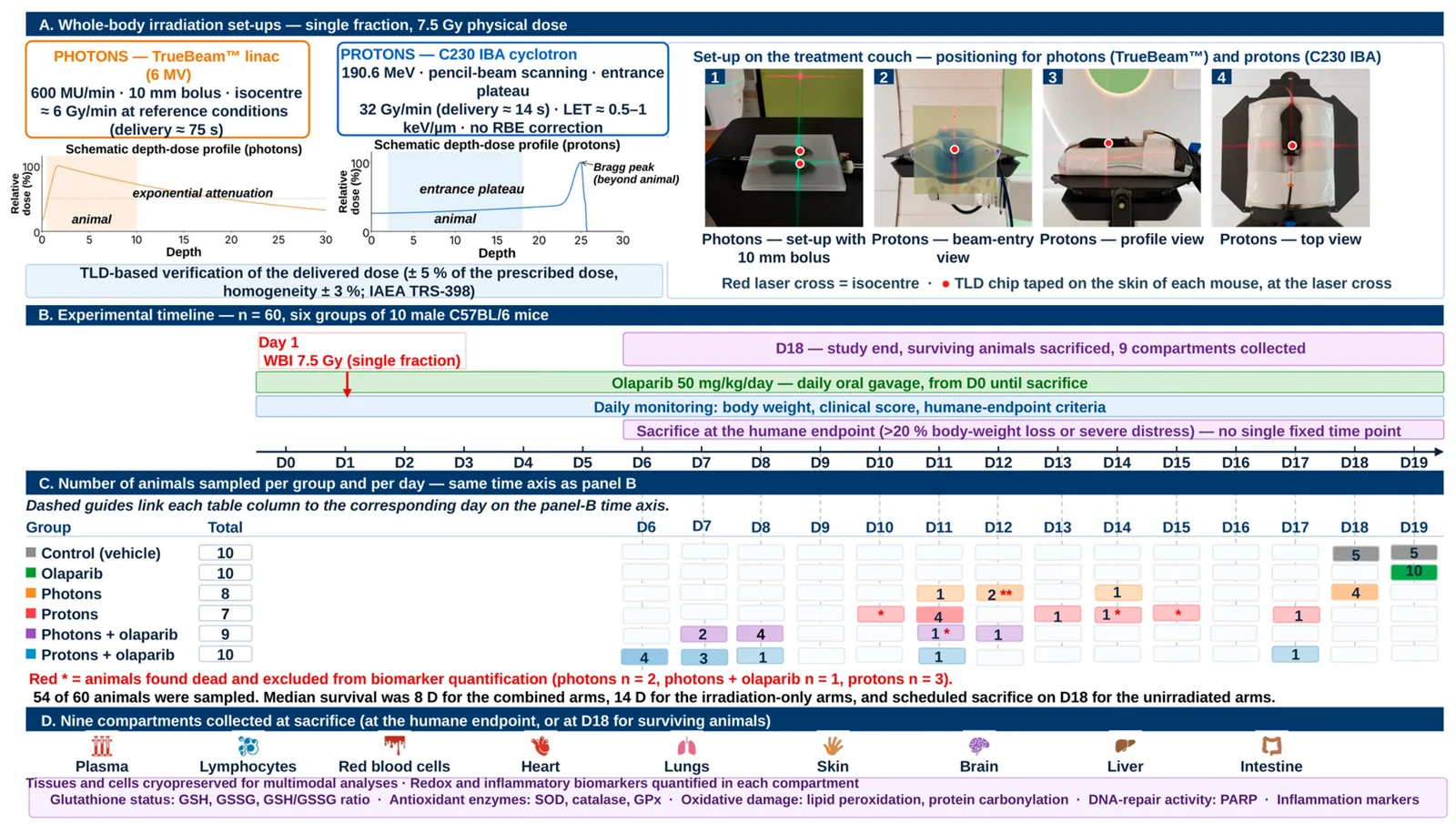
Experimental design: irradiation set-ups, timeline, sacrifice distribution, and multi-compartment sampling. (**A**) Irradiation. Photon WBI: TrueBeam^TM^ (Varian Medical Systems, Palo Alto, CA, USA) linac (6 MV, ≈6 Gy/min, ≈75 s for 7.5 Gy, 10 mm bolus). Proton WBI: C230 IBA (IBA, Ion Beam Applications, Louvain-la-Neuve, Belgium) cyclotron (Institut Curie, Orsay), 190.6 MeV pencil-beam scanning (32 Gy/min, ≈14 s), animals in the entrance plateau upstream of the Bragg peak (LET ≈ 0.5–1 keV/µm, RBE = 1). Red dots, TLD chip at the isocentre; dose verified within ± 5% (± 3% homogeneity; IAEA TRS-398). (**B**) Timeline. Sixty male C57BL/6 mice, six groups of ten. Olaparib (50 mg/kg/day) by daily gavage from D0 until sacrifice; single 7.5 Gy WBI at D1. Animals were sacrificed at the humane endpoint or at D18 for survivors. (**C**) Sacrifice distribution. Animals sampled per group and per day; red asterisks, animals found dead and excluded from biomarker quantification. Most animals in the combination arms reached the humane endpoint between D7 and D10 (median day 8) and in the irradiation-only arms between D10 and D15; surviving animals in all irradiated arms, together with the unirradiated arms, were sacrificed at D18. (**D**) Sampling. Nine compartments (plasma, lymphocytes, erythrocytes, heart, lungs, skin, brain, liver, intestine) were collected at sacrifice for redox and inflammatory biomarkers. Abbreviations: WBI, whole-body irradiation; LET, linear energy transfer; RBE, relative biological effectiveness; TLD, thermoluminescent dosimeter.

Animals were anesthetized with isoflurane in a 50% room air/50% oxygen mixture, with identical anesthesia and positioning conditions across all groups, minimizing differential effects on redox biomarkers [36]. Olaparib was administered by oral gavage at 50 mg/kg once daily, beginning 24 h before irradiation (day 0) and continued every day thereafter until sacrifice [37,38] (Figure 1). 

### 2.2. Follow-Up and Biomarker Quantification

**Figure 2:**
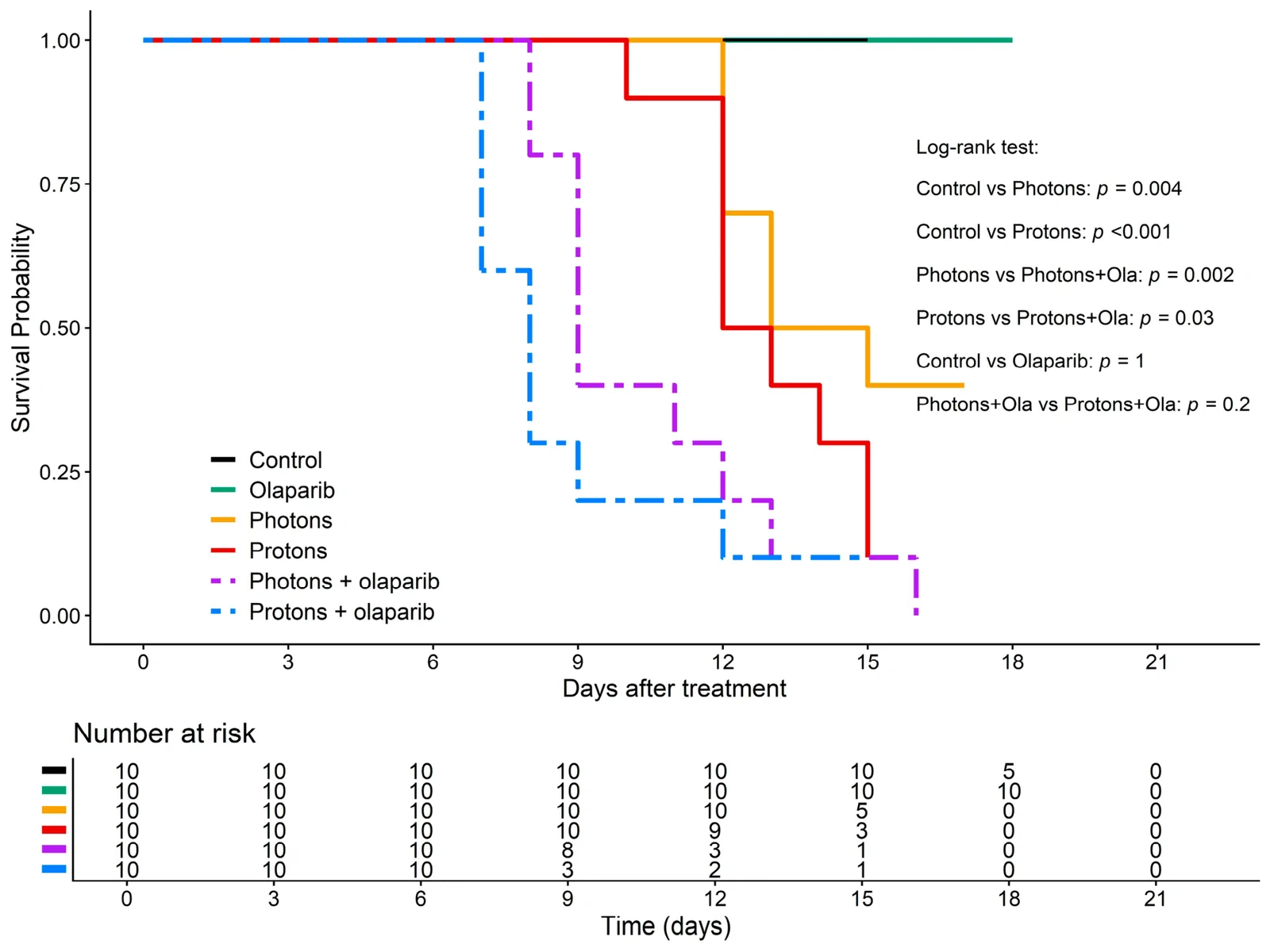
Kaplan–Meier survival curves for the six treatment groups over the 18-day observation period. The analysis includes all 60 randomized animals (n = 10 per group); the number at risk is shown below the plot. The six animals excluded from biomarker analysis (photons, n = 2; photons + olaparib, n = 1; protons, n = 3) are all included here as observed deaths. Control and olaparib-alone animals maintained 100% survival and were electively sacrificed at day 18. Median survival was 14 days after photons and 12.5 days after protons, falling to 9 and 8 days, respectively, with olaparib. Pairwise log-rank (Mantel–Cox) *p*-values are displayed on the plot: control vs. olaparib, *p* = 1 (q = 1); control vs. photons, *p* = 0.004 (q = 0.008); control vs. protons, *p* < 0.001 (q < 0.001); photons vs. photons + olaparib, *p* = 0.002 (q = 0.004); protons vs. protons + olaparib, *p* = 0.03 (q = 0.046); photons + olaparib vs. protons + olaparib, *p* = 0.2 (q = 0.33). Benjamini–Hochberg correction was applied across the six pre-specified pairwise comparisons. Survival restricted to the 54 animals retained for biomarker analysis is shown in Supplementary Figure S3.

The follow-up period was designed to match that of the earlier dose-titration study [26], and the project authorization provided for a follow-up of up to 12 weeks. Mortality, however, greatly exceeded expectations in all irradiated arms, and the addition of olaparib rendered the exposure near-lethal in both modalities. All irradiated arms met their predefined humane endpoint well before that time and were sampled accordingly. The control and olaparib-alone arms showed no radiation-related mortality and could have been followed until 12 weeks but were electively sacrificed at day 18 so that all compartments were harvested within a comparable observation window. From the day of irradiation onward, animals were monitored five days per week against the standardized CEEA-IC/SBEA-IC rodent clinical scoring grid, a binary (present/absent) checklist assessing external appearance (posture, coat condition, abdominal distension, pallor, fecal status, self-mutilation) and handling response (reluctance to move, righting reflex), as prescribed by the approved project authorization. Animals reaching the grid-defined threshold—four minor criteria or a single major criterion (impaired mobility, laboured breathing, or >20% body-weight loss)—were immediately euthanized [39,40,41,42]. Humane endpoints were applied individually; no group-level stopping rule was predefined. Median time to biomarker sampling in the 54 animals analyzed was 18 days in the unirradiated arms, 16 days for photons, 12 days for protons in the irradiation-only arms, and 8 days in both combination arms (Supplementary Figure S1). The observation window captured the acute radiation-induced toxicity phase and the initial transition toward late radiation-related changes. Only animals alive at their endpoint were sampled for biomarker quantification. Six animals could not be included in the biomarker analysis because they were found dead before the scheduled sacrifice (photons, *n* = 2; photons plus olaparib, *n* = 1; protons, *n* = 3) and had an uncertain postmortem interval. For each of the six animals found dead, the records confirmed that death had occurred less than 24 h after the last observation at which the animal was alive. Effective biomarker sample sizes were therefore n = 10 (control), 10 (olaparib), 8 (photons), 9 (photons + olaparib), 7 (protons), and 10 (protons + olaparib). All six animals were nevertheless retained in the survival analysis as observed deaths, which was therefore performed on all 60 randomized animals (Figure 2); survival of the 54-animal biomarker cohort is shown in Supplementary Figure S3. Blood was collected by cardiac puncture (for plasma, erythrocytes, and lymphocytes), and organs (brain, heart, liver, lung, intestine, and thoracic skin) were snap-frozen, pulverized, and homogenized.

In total, 74 biomarkers representing 25 distinct biological entities were quantified across nine compartments (brain, heart, liver, lung, intestine, thoracic skin, lymphocytes, erythrocytes, and plasma), grouped into six categories: (1) oxidative and DNA damage—protein carbonylation (DNPH method) and lipid peroxidation (TBARS/MDA), plus DNA oxidation (8-OXO-dG; ELISA, Abcam) in plasma and lymphocytes only; (2) antioxidant enzyme activities—superoxide dismutase (Flohé and Ötting method), catalase (Aebi method), and glutathione peroxidase (Paglia method); (3) glutathione redox status—reduced (GSH) and oxidized (GSSG) glutathione and the GSH/GSSG ratio (enzymatic recycling); (4) total protein content (Bradford assay); (5) DNA repair activity—PARP activity, measured as nicotinamide released upon NAD^+^ cleavage during PARP-mediated poly-ADP-ribosylation; and (6) inflammatory mediators—TGF-β1 and 13 cytokines (IL-1α, IL-1β, IL-6, IL-10, IL-12p70, IL-17A, IL-23, IL-27, IFN-γ, IFN-β, TNF-α, MCP-1, and GM-CSF) quantified by multiplex bead assay (LEGENDplex™/MILLIPLEX^®^) in plasma only (Supplementary Table S2). Most protocols were performed as previously described by Chaouni et al. [26,43]. Total protein content was measured by the Bradford assay and is expressed per gram of wet tissue (mg/g tissue) for organs and per millilitre (mg/mL) for plasma, erythrocytes, and lymphocytes; protein-dependent biomarkers are expressed per mg of total protein.

*Reagents*

When not specified in the text, chemicals were purchased from Sigma-Aldrich (Saint-Louis, MO, USA).

### 2.3. Statistical Analyses

Data are expressed as mean ± SD, and effect sizes as Cohen’s d. Because sacrifice timing differed between arms (median 18, 14, and 8 days in the unirradiated, irradiation-only, and combination arms, respectively), biomarkers were compared directly only between the arms with the closest sampling times; all other contrasts were adjusted for the day of sacrifice. Six pairwise comparisons were pre-specified. The principal pre-specified comparison, photons + olaparib versus protons + olaparib, was analyzed using a two-sided Wilcoxon–Mann–Whitney rank-sum test. This contrast was designated as the principal comparison because the two arms were the closest in sampling time of all irradiated pairs, with animals being sampled at individual, endpoint-driven times with a median of day 8 in both arms. The analysis is not adjusted for the individual day of sacrifice, and the residual difference in sampling times is addressed in the Limitations Section. Control versus olaparib alone, with both arms being electively sacrificed on day 18, is the only genuinely time-matched pair. The four remaining comparisons involved arms sampled at different times and were therefore treated as exploratory and analyzed by rank-based analysis of covariance (ANCOVA) adjusting for the day of sacrifice: (3) control versus photon irradiation, (4) control versus proton irradiation, (5) photon irradiation versus photons + olaparib, and (6) proton irradiation versus protons + olaparib. To explore the relationship between biomarker values and sampling time, individual values were plotted against the actual day of sacrifice for biomarkers showing at least one FDR-significant day-adjusted exploratory comparison (Supplementary Figure S2).

Benjamini–Hochberg false discovery rate (FDR) correction was applied within each biological compartment for each comparison, and findings are reported as FDR-significant (q < 0.05) or, otherwise, as exploratory nominal signals (*p* < 0.05). For the four time-shifted comparisons, significance was based on rank-based ANCOVA adjusted for the day of sacrifice, with Benjamini–Hochberg FDR correction applied within each biological compartment. These day-adjusted results are reported in Supplementary Tables S4–S10 and summarized in Supplementary Table S11. Survival was estimated by the Kaplan–Meier method and compared using log-rank tests with FDR correction for the multiple pairwise contrasts. All analyses were performed in R (v4.x).

## 3. Results

### 3.1. Survival and Body Weight

**Figure 3:**
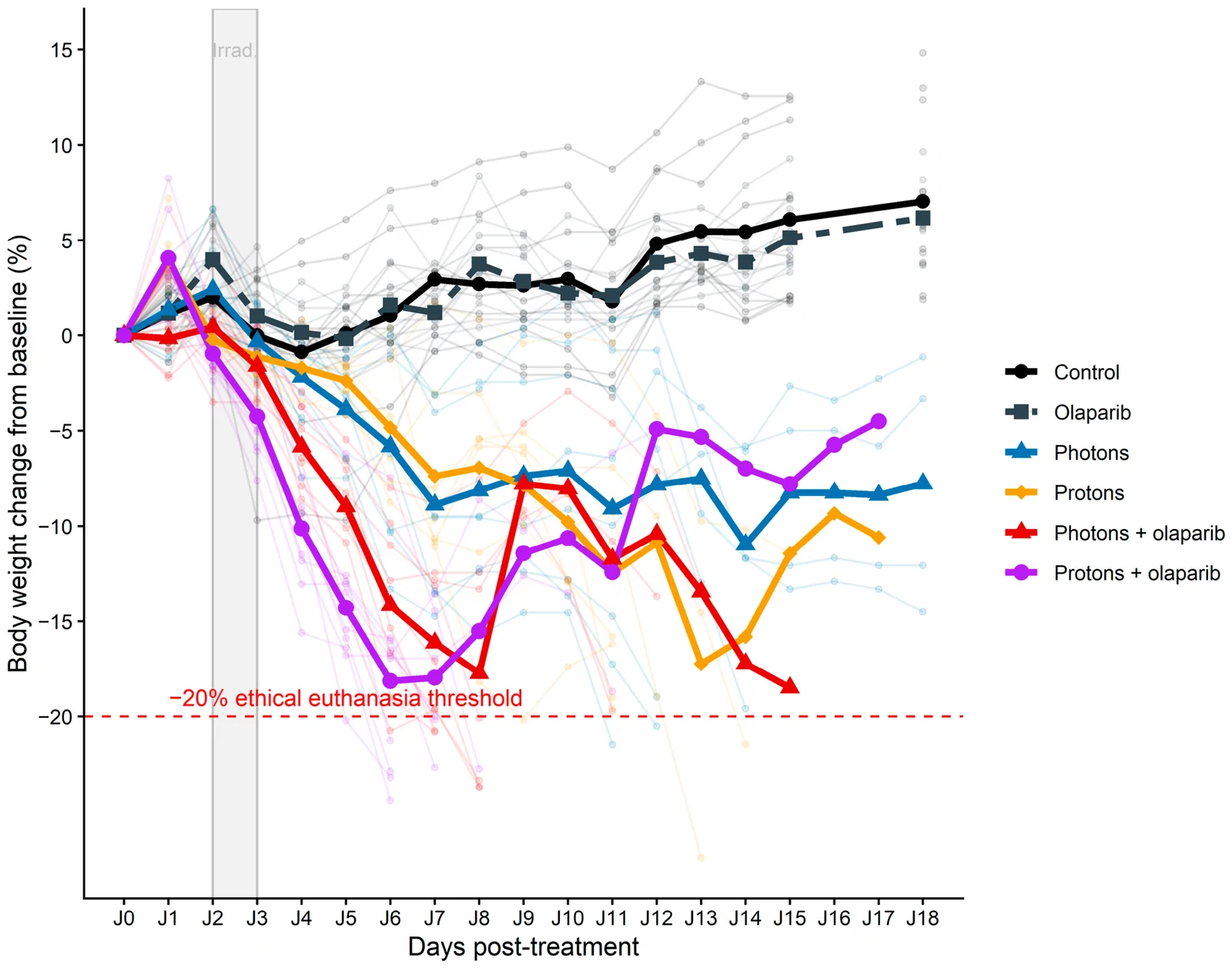
Body-weight change from baseline over the observation period for the six treatment groups. Thin lines show individual animals and thick lines show the group means; the horizontal dashed line marks the −20% body-weight-loss ethical euthanasia threshold. Group sizes were *n* = 10 (control), 10 (olaparib), 8 (photons), 7 (protons), 9 (photons + olaparib), and 10 (protons + olaparib). Control and olaparib-alone animals gained weight throughout (+7.0 ± 4.7% and +6.2 ± 1.9% at day 18, respectively), whereas all irradiated arms lost weight. Weight loss was intermediate after single-modality irradiation (photons −13.4 ± 7.5%; protons −17.7 ± 5.1%) and most severe in the combination arms, which reached the euthanasia threshold earliest (maximum loss −21.3 ± 2.3% for photons + olaparib and −19.6 ± 4.0% for protons + olaparib). Curves are truncated at each group’s median time to sacrifice, beyond which surviving-animal numbers become too small for reliable group means.

Survival was analyzed in all 60 randomized animals. Control and olaparib-alone groups maintained 100% survival with positive weight gain at day 18 (+7.0 ± 4.7% and +6.2 ± 1.9%, respectively). Single-modality irradiation yielded intermediate outcomes: median survival was 14 days after photons (6/10 deaths, 40% survival at day 18, −13.4 ± 7.5% weight loss) and 12.5 days after protons (9/10 deaths, 10% survival, −17.7 ± 5.1%). Both combination arms showed markedly increased toxicity, with median survival falling to 9 days for photons + olaparib (10/10 deaths, 0% survival at day 18, maximum weight loss −21.3 ± 2.3%) and 8 days for protons + olaparib (9/10 deaths, 10% survival, −19.6 ± 4.0%), with deaths occurring between days 7 and 15 (Figure 2 and Figure 3). Olaparib significantly worsened survival relative to irradiation alone in both modalities (photons: *p* = 0.002, q = 0.004; protons: *p* = 0.03, q = 0.046, log-rank with Benjamini–Hochberg correction across the six pre-specified pairwise comparisons). No significant difference was observed between the two combination arms (*p* = 0.2, q = 0.33) or between the two irradiation-only arms (protons vs. photons, *p* = 0.2). Restricting the analysis to the 54 animals sampled for biomarkers gave consistent results (Supplementary Figure S3).

### 3.2. Olaparib Monotherapy

Olaparib alone nominally modified 14 of 74 biomarkers (18.9%, *p* < 0.05), of which 5 remained significant after FDR correction (*q* < 0.05). The most robust hepatic effects were protein accumulation (+52.4%, *p* < 0.001, *q* = 0.022) and a marked reduction in hepatic PARP enzymatic activity (−43.0%, *p* = 0.002, *q* = 0.033), with the latter confirming effective target engagement and impaired DNA damage response capacity. In the brain, a paradoxical SOD upregulation (+56.7%, *p* = 0.002, *q* = 0.037) suggested a possible adaptive redox response in the absence of genotoxic stress—a baseline against which radiation combinations must be interpreted. Olaparib also depleted erythrocyte antioxidant enzymes (catalase −54.0%, *q* = 0.022; glutathione peroxidase −73.8%, *q* = 0.029), indicating a systemic reduction in circulating antioxidant capacity. A decrease in hepatic lipid peroxidation (−22.7%, *p* = 0.019), consistent with NAD^+^ preservation from PARP1 inhibition, was also observed but did not survive FDR correction (*q* = 0.150). Plasma cytokine levels, including TGF-β1, did not differ significantly between control and olaparib-alone animals (Supplementary Table S3), providing no evidence of a systemic inflammatory response to olaparib alone at this time point.

### 3.3. Cerebral and Cardiac Biomarkers

**Figure 4:**
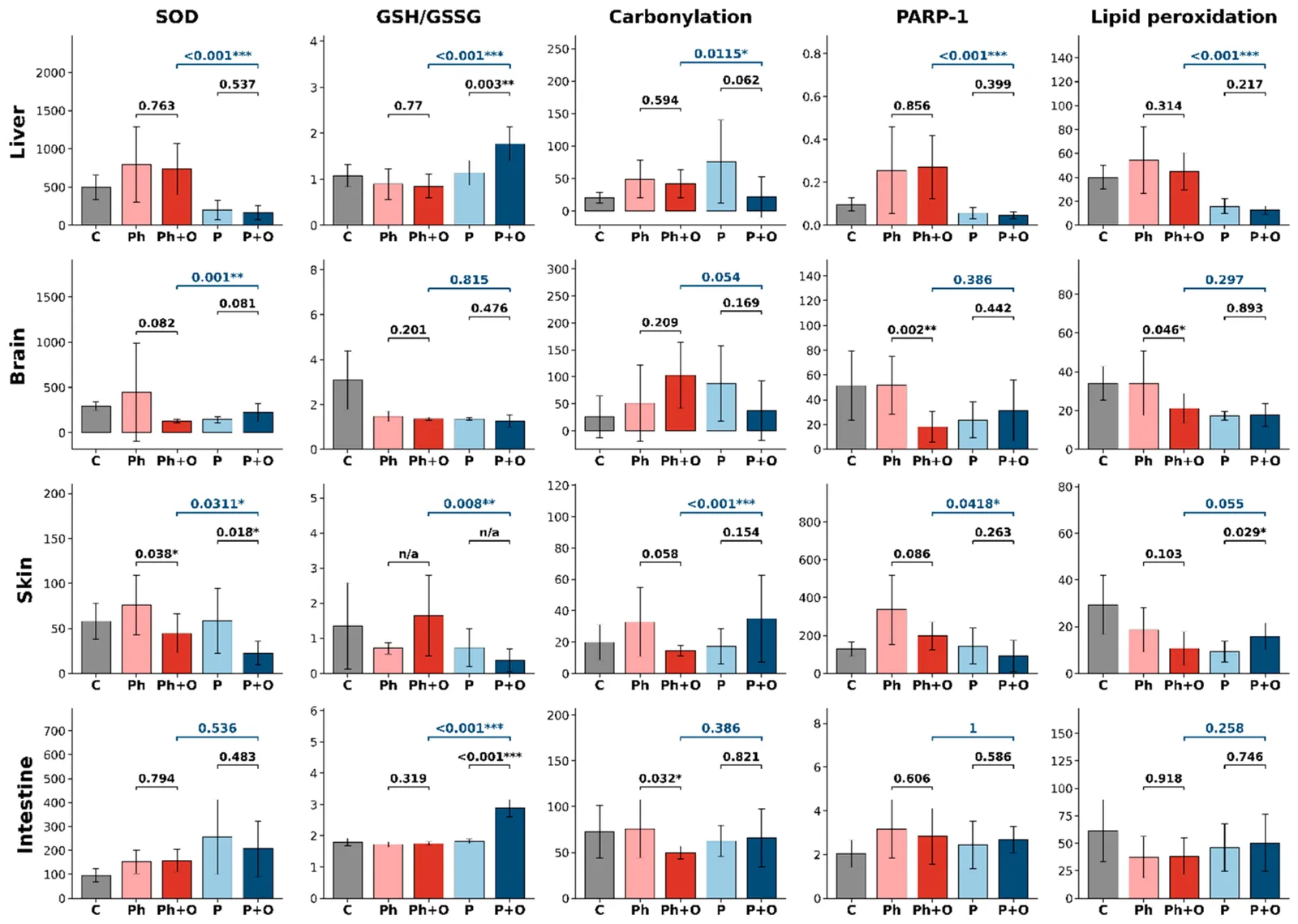
Selected antioxidant, PARP-1, and lipid peroxidation biomarkers in liver, brain, skin, and intestine. Bars are group mean + SD. Rows are tissues; columns are SOD, GSH/GSSG ratio, protein carbonylation, PARP-1, and lipid peroxidation (MDA/TBARS). C, control; Ph, photons; Ph+O, photons + olaparib; P, protons; P + O, protons + olaparib. Blue bracket: principal comparison (P + O vs. Ph+O, day 8), Wilcoxon rank-sum test. Black brackets: exploratory comparisons (Ph vs. Ph + O; P vs. P+O), day-adjusted ANCOVA. * *p* < 0.05, ** *p* < 0.01, *** *p* < 0.001; n/a, model not fitted (missing data). Abbreviations: SOD, superoxide dismutase; GSH/GSSG, reduced/oxidized glutathione; PARP-1, poly(ADP-ribose) polymerase-1; MDA/TBARS, malondialdehyde/thiobarbituric acid reactive substances; ANCOVA, analysis of covariance.

In cerebral tissue, day-adjusted analysis showed that adding olaparib to photon irradiation significantly impaired antioxidant and repair systems relative to photons alone (Supplementary Figure S4): glutathione peroxidase (−79.5%, *p* = 0.002, *q* = 0.010), PARP1 activity (−65.1%, *p* = 0.002, *q* = 0.010), and catalase (−49.0%, *p* = 0.011, *q* = 0.037) were all reduced after FDR correction, together with a nominal decrease in lipid peroxidation (−38.2%, *p* = 0.046). The SOD reduction and protein accumulation seen on unadjusted testing did not persist after adjustment for the day of sacrifice (both *p* > 0.08). In contrast, adding olaparib to proton irradiation tended to raise cerebral catalase (+55.9%, *p* = 0.027) and SOD (+54.0%, *p* = 0.081), with neither surviving FDR correction. This directional pattern resembled that seen under olaparib monotherapy. In the principal inter-arm comparison, cerebral SOD activity was 75.1% higher under protons + olaparib than under photons + olaparib (*p* = 0.001, q = 0.010), with a non-significant trend toward lower protein carbonylation (−63.6%, *p* = 0.054); SOD was the only cerebral biomarker differing between the two combination arms after FDR correction (Table 1, Figure 4; Supplementary Table S4, Supplementary Figure S4).

In cardiac tissue, the pronounced increases in antioxidant enzymes and glutathione (catalase, SOD, GPx, and GSH) apparent after photons + olaparib on unadjusted testing did not survive adjustment for the day of sacrifice. After day-adjusted FDR correction, photons + olaparib was instead characterized, relative to photons alone, by a shift toward a more oxidized cardiac glutathione pool (GSH/GSSG −26.1%, *p* = 0.002, *q* = 0.010) and a marked reduction in total protein (−50.6%, *p* = 0.001, *q* = 0.010), with only nominal increases in oxidized glutathione (GSSG +288.8%, *p* = 0.031) and PARP1 activity (+145.3%, *p* = 0.025). Proton irradiation alone produced a nominal increase in cardiac protein carbonylation (+223.5%, *p* = 0.021) that did not survive FDR correction, whereas photons alone did not, suggesting a modality-specific oxidative signature. No cardiac biomarker differed significantly between the two combination arms in the principal comparison (Supplementary Table S5).

### 3.4. Hepatic, Intestinal, Cutaneous, and Pulmonary Biomarkers

The liver showed the largest number of FDR-significant biomarker differences between the two combination-treatment groups. The most pronounced finding was the difference in hepatic total protein concentration, which was higher in the protons + olaparib group than in the photons + olaparib group (+344.9%; *p* < 0.001, q < 0.001). Hepatic total protein followed opposite directions in the two modalities. Relative to control, it was reduced after photon irradiation alone (−32.2%, q = 0.007) and was close to control values under photons + olaparib, whereas it was markedly increased after proton irradiation alone (+176.6%, q < 0.001) and numerically further under protons + olaparib (+30.4% vs. protons alone, q = 0.15; Supplementary Table S6). The divergence between the combination arms therefore reflects a modality-dependent deviation from the unirradiated baseline, already present without olaparib and confined to the proton arms. No similar difference in total protein concentration was observed in the other tissue compartments. Against this background, the protons + olaparib group showed lower values for all protein-normalized hepatic biomarkers: lipid peroxidation (−71.9%; *p* < 0.001, *q* < 0.001), protein carbonylation (−48.7%; *p* = 0.012, *q* = 0.013), PARP1 activity (−82.9%; *p* < 0.001, *q* < 0.001), catalase activity (−87.5%; *p* < 0.001, *q* < 0.001), and SOD activity (−77.8%; *p* < 0.001, *q* < 0.001) (Supplementary Table S6). Because these biomarkers were normalized to protein content, their lower values may partly reflect the difference in the denominator and should not, by themselves, be interpreted as evidence of reduced hepatic oxidative damage. By contrast, the GSH/GSSG ratio, calculated as the quotient of two quantities normalized using the same denominator, was mathematically unaffected by protein normalization and was higher in the protons + olaparib group (+108.2%; *p* < 0.001, *q* < 0.001). Moreover, conventional markers of liver toxicity—including AST, ALT, bilirubin, histopathological changes, and fibrosis markers—were not assessed. Therefore, the observed differences in oxidative stress biomarkers cannot be interpreted as evidence of hepatoprotection or reduced liver injury; they reflect only the redox and antioxidant parameters measured in this study.

In the intestine, proton irradiation alone was associated with higher SOD and catalase activity relative to control (SOD +166.7%, q = 0.020; catalase +44.0%, q < 0.001), which olaparib did not further modify in either modality. In the principal inter-arm comparison, glutathione peroxidase was higher under protons + olaparib (+449.9%, *p* = 0.014, q = 0.047), together with lower GSSG (−42.7%, q = 0.003) and a higher GSH/GSSG ratio (+64.1%, q < 0.001). Adding olaparib to protons also lowered GSSG (−45.1%, q < 0.001) and raised the GSH/GSSG ratio (+58.3%, q < 0.001), a change not observed when olaparib was added to photons (Supplementary Table S7).

On direct inter-arm comparison, thoracic skin showed the most divergent oxidative stress responses between the two combination arms. Relative to photons + olaparib, protons + olaparib was associated with a lower GSH/GSSG ratio (−77.6%, *p* = 0.008, q = 0.036) and higher protein carbonylation (+142.9%, *p* < 0.001, q = 0.002), both surviving FDR correction, together with near-complete GSH depletion (−95.7%, *p* = 0.023, q = 0.083), reduced SOD (−49.1%, *p* = 0.031, q = 0.083), and lower PARP1 activity (−53.8%, *p* = 0.042, q = 0.084), which reached nominal significance only. This pattern—impaired antioxidant capacity together with elevated protein carbonylation and lipid peroxidation—is consistent with profiles associated with delayed wound healing and exacerbated radiation dermatitis [44,45]. In the day-adjusted (ANCOVA) within-modality analyses, protons + olaparib versus protons alone showed increased LPO (+71.5%, *p* = 0.029) and reduced SOD (−61.3%, *p* = 0.018), while photons + olaparib versus photons alone showed reduced SOD (−41.7%, *p* = 0.038); these directional changes are consistent with the same profile but should be interpreted as trends, as none survived FDR correction (Supplementary Table S8). Taken together, these findings identify thoracic skin as the compartment in which the oxidative biomarker differences between the two combination arms were largest, with protons + olaparib showing lower antioxidant capacity and higher protein carbonylation—a hypothesis-generating observation that requires confirmation by histological or clinical dermatitis assessment (see Section 4).

Pulmonary biomarkers did not differ significantly between the two combination arms (all NS; protons + olaparib vs. photons + olaparib). Within the proton arm, lipid peroxidation was selectively higher under protons + olaparib than under protons alone (+57.0%, *p* = 0.029), although this difference did not survive FDR correction and should be regarded as a trend only (Supplementary Table S9).

### 3.5. Systemic and Blood Biomarkers

Olaparib alone did not alter blood cytokine levels relative to controls (Supplementary Table S3). Conversely, both irradiation modalities induced marked systemic inflammation. In the day-adjusted (ANCOVA) analyses versus control, MCP-1 was the only cytokine significantly elevated under both modalities (≈×19 under photons and ≈×18 under protons, both q < 0.05). The remaining responses were modality-dependent: IL-17A, IL-1β, and IL-27 reached FDR significance under photons only, and IL-6 (≈×15) under protons only, whereas the increases in IFN-γ, IL-10, and TNF-α did not survive adjustment for the day of sacrifice in either modality. This inflammatory response was accompanied by paradoxical plasma TGF-β1 depletion (photons −89.4%, protons −98.8%, both *p* < 0.001, q < 0.01). Olaparib introduced modality-specific perturbations. In the principal inter-arm comparison, MCP-1 was 69.5% lower under protons + olaparib than under photons + olaparib (d = −0.78, *p* = 0.043), although this difference did not survive FDR correction (q = 0.57). In the day-adjusted within-modality analysis, plasma TGF-β1 was partially recovered under protons + olaparib relative to protons alone (+521.2%, *p* = 0.024, q = 0.072), reaching approximately 7% of control levels, whereas no such recovery was observed under photons + olaparib; this nominal difference did not survive FDR correction. In erythrocytes, the principal inter-arm comparison showed higher GSH (+56.9%, *p* = 0.008, q = 0.027), GSSG (+53.3%, *p* = 0.003, q = 0.021), and SOD (+80.8%, *p* = 0.017, q = 0.040) under protons + olaparib than under photons + olaparib, all surviving FDR correction (Supplementary Table S10).

### 3.6. Global Summary

Direct comparison of protons + olaparib versus photons + olaparib (principal comparison) identified 23/74 biomarkers (31.1%) with nominal between-arm differences, 18 of which survived FDR correction (Table 1). Seven of these 18 were hepatic measurements expressed per mg of total protein (lipid peroxidation, GSH, GSSG, protein carbonylation, PARP activity, catalase, and SOD). Because total hepatic protein itself differed 4.4-fold between the two arms (+344.9%), these seven measurements depend on the normalization and were considered non-interpretable; they are shown in Table 1 for transparency only. The remaining 11 FDR-significant differences were interpretable: the hepatic GSH/GSSG ratio (invariant to the normalization) and hepatic total protein, together with intestinal, cerebral, erythrocyte, and cutaneous measurements. Under protons + olaparib relative to photons + olaparib, nine of these 11 were higher (hepatic GSH/GSSG ratio and total protein; cerebral SOD; intestinal GPx and GSH/GSSG ratio; erythrocyte GSH, GSSG and SOD; cutaneous protein carbonylation) and two were lower (intestinal GSSG and cutaneous GSH/GSSG ratio). These differences are descriptive and do not indicate which combination is less toxic.

**Table 1:** Biomarkers differing between protons + olaparib and photons + olaparib in the principal inter-arm comparison.

Biomarker	Control M (SD) (N = 10)	Pro + Ola M (SD) (N = 10)	Ph + Ola M (SD) (N = 9)	Δ (%)	*p*-Value	Cohen’s d	Direction (Pro + Ola)	FDRq
LIVER—not dependent on protein normalization (interpretable)								
GSH/GSSG (ratio)	1.08 (0.24)	1.77 (0.37)	0.85 (0.26)	+108.2%	<0.001	2.89	*** Higher	<0.001
Protein	12.5 (2.06)	45.2 (8.99)	10.2 (2.94)	+344.9%	<0.001	5.11	*** Higher	<0.001
LIVER—dependent on protein normalization (non-interpretable)								
LPO	40.1 (9.99)	12.7 (3.34)	45.2 (15.4)	−71.9%	<0.001	−3.00	*** † Lower	<0.001
GSH	105 (43.8)	17.0 (8.25)	74.2 (60.9)	−77.1%	<0.001	−1.36	*** † Lower	<0.001
GSSG	95.9 (31.3)	9.84 (4.91)	92.4 (65.1)	−89.3%	<0.001	−1.85	*** † Lower	<0.001
Protein carbonylation	20.5 (8.27)	21.4 (31.6)	41.7 (21.7)	−48.7%	0.012	−0.73	* † Lower	0.013
PARP activity	0.097 (0.030)	0.047 (0.018)	0.272 (0.147)	−82.9%	<0.001	−2.23	*** † Lower	<0.001
Catalase activity	374 (402)	10.9 (6.95)	87.5 (63.9)	−87.5%	<0.001	−1.74	*** † Lower	<0.001
SOD activity	497 (157)	163 (92.4)	735 (335)	−77.8%	<0.001	−2.40	*** † Lower	<0.001
BRAIN								
SOD activity	293 (45.9)	222 (99.7)	127 (20.8)	+75.1%	0.001	1.28	** Higher	0.010
Protein carbonylation	25.4 (38.9)	37.4 (55.2)	103 (61.4)	−63.6%	0.054	−1.12	Lower	0.270
INTESTINE								
GPx activity	0.99 (0.61)	2.71 (2.55)	0.49 (0.41)	+449.9%	0.014	1.21	* Higher	0.047
GSSG	126 (28.1)	87.4 (26.2)	152 (38.4)	−42.7%	<0.001	−1.98	** Lower	0.003
GSH/GSSG (ratio)	1.80 (0.12)	2.88 (0.27)	1.75 (0.06)	+64.1%	<0.001	5.73	*** Higher	<0.001
SKIN								
GSH	10.4 (9.66)	0.17 (0.98)	3.93 (4.59)	−95.7%	0.023	−1.22	Lower	0.083
GSH/GSSG (ratio)	1.35 (1.23)	0.37 (0.32)	1.65 (1.15)	−77.6%	0.008	−1.52	* Lower	0.036
Protein carbonylation	19.8 (11.2)	34.9 (27.8)	14.4 (3.24)	+142.9%	<0.001	0.97	** Higher	0.002
SOD activity	58.0 (20.2)	22.6 (13.0)	44.4 (21.6)	−49.1%	0.031	−1.27	Lower	0.083
PARP activity	129 (38.5)	91.3 (82.6)	198 (72.5)	−53.8%	0.042	−1.36	Lower	0.084
ERYTHROCYTES								
GSH	39.8 (13.6)	29.0 (7.25)	18.5 (4.11)	+56.9%	0.008	1.76	* Higher	0.027
GSSG	10.3 (3.27)	9.76 (2.31)	6.36 (1.42)	+53.3%	0.003	1.75	* Higher	0.021
SOD activity	27.8 (11.2)	36.2 (15.7)	20.0 (3.62)	+80.8%	0.017	1.38	* Higher	0.040
SYSTEMIC (Plasma)								
MCP-1	9.81 (6.51)	108 (101)	354 (447)	−69.5%	0.043	−0.78	Lower	0.566

Note. Biomarkers showing a nominal between-arm difference in the principal head-to-head comparison of the two combination arms (protons + olaparib vs. photons + olaparib), analyzed by a two-sided Wilcoxon–Mann–Whitney rank-sum test. Median sacrifice was day 8 in both arms with no significant difference in survival (log-rank *p* = 0.2, q = 0.33). Values are group means and standard deviations (M, SD) for control, protons + olaparib, and photons + olaparib. Δ(%) is the change in protons + olaparib relative to photons + olaparib; Cohen’s d is the standardized effect size; *p* is the unadjusted Wilcoxon *p*-value; FDR q is its Benjamini–Hochberg-adjusted value computed within each tissue compartment. Direction indicates whether the value is higher or lower in Pro + Ola than in Ph + Ola; an asterisk (*) denotes a difference maintained after FDR correction (q < 0.05). Significance: * q < 0.05; ** q < 0.01; *** q < 0.001. Abbreviations: GPx, glutathione peroxidase; GSH, reduced glutathione; GSSG, oxidized glutathione; LPO, lipid peroxidation; MCP-1, monocyte chemoattractant protein-1; PARP, poly(ADP-ribose) polymerase; SOD, superoxide dismutase. † Hepatic biomarkers are expressed per mg of total protein. Total hepatic protein content differed between the two combination arms (+344.9%, q < 0.001), a difference observed in no other compartment; the direction of these findings cannot therefore be established with confidence.

## 4. Discussion

To our knowledge, this is the first in vivo study to characterize normal-tissue oxidative stress and antioxidant responses to a PARP inhibitor combined with whole-body proton versus photon irradiation. Existing evidence on PARPi–proton combinations remains limited, consisting predominantly of in vitro radiosensitization studies [19,20], with only sparse in vivo tumour-model data and virtually no dedicated normal-tissue toxicity studies. A recently reported clinical case of severe radiation necrosis following proton therapy combined with the PARP inhibitor veliparib further highlighted the potential for enhanced normal-tissue effects and the need for dedicated preclinical toxicity studies [46].

Proton and photon irradiation are conventionally regarded as radiobiologically equivalent through the application of a single, spatially invariant relative biological effectiveness (RBE) of 1.1 for protons relative to high-energy photons. This convention, however, increasingly conflicts with experimental and modelling evidence that proton RBE is not constant but varies with linear energy transfer (LET), dose per fraction, tissue α/β, and biological endpoint, rising from ≈1.1 in the entrance plateau to ≈1.3 at the distal edge and ≈1.7 in the distal fall-off [18,47]. These deviations have become clinically salient with the widespread adoption of pencil-beam scanning (PBS), whose sharp dose conformity places high-LET track-ends within or adjacent to organs at risk, locally elevating the biologically effective dose above that predicted by a fixed RBE of 1.1 [48,49]. Against this background, the assumption that photon and proton exposures produce interchangeable normal-tissue effects is a simplification, and the modality-dependent oxidative and antioxidant responses reported here provide converging biological evidence that radiation quality—beyond absorbed dose and generic RBE—shapes normal-tissue outcome [50].

This exploratory study provides preclinical, multi-organ data showing modality-associated differences in tissue redox biomarkers in mice receiving olaparib and near-lethal whole-body irradiation. PARP inhibition may exert opposing effects on irradiated normal tissue. Given concomitantly, olaparib impairs single-strand break repair and traps PARP-1 on chromatin, converting radiation-induced lesions into double-strand breaks and radiosensitizing the tissue [7,8]; given after exposure, it may be protective by limiting NAD^+^ depletion and inflammatory signalling [28,51], as shown by the attenuation of hematopoietic and oxidative injury after a single post-exposure dose in mice receiving 2.5 Gy [27]. In the present study, concomitant olaparib shortened survival with both modalities, and survival did not differ between the combination arms. Against this common background, higher values under protons + olaparib were observed in the brain (SOD), intestine (GPx, GSH/GSSG ratio), and erythrocytes (GSH, GSSG, SOD), together with a higher hepatic GSH/GSSG ratio, which is the only hepatic redox measure unaffected by protein normalization. In the skin, the GSH/GSSG ratio was lower and protein carbonylation higher under protons + olaparib. No difference survived FDR correction in heart, lung, lymphocytes, or plasma. These observations raise the hypothesis that radiation quality may influence tissue redox status during PARP inhibition; this requires dedicated testing. Obtained at near-lethal doses in a whole-body model, they do not establish a difference in normal-tissue toxicity; this will require dedicated studies using localized, fractionated irradiation and histological and functional endpoints.

In the brain, adding olaparib to photon irradiation was associated with lower GPx (−79.5%, q = 0.010), catalase (−49.0%, q = 0.037), and PARP1 activity (−65.1%, q = 0.010) than photons alone (day-adjusted analysis). The lower PARP1 activity is compatible with blood–brain barrier penetration by olaparib [52]; its functional significance was not assessed, although PARP1 has roles in synaptic plasticity and neuroprotection [53,54]. By contrast, adding olaparib to protons was associated with higher cerebral antioxidant enzyme activity, which reached nominal significance only for catalase (+55.9%, *p* = 0.027, q = 0.27) and not for SOD (+54.0%, *p* = 0.081); GPx and PARP1 changed in the same direction without significance (Supplementary Figure S4). These within-modality contrasts are exploratory and the mechanisms were not tested. A hormesis-like adaptive response involving Nrf2/ARE activation [55,56,57] is one possible explanation, but no Nrf2 or serial biochemical data are available, so it remains speculative. An alternative explanation—that this divergence simply reflects differential survivor bias between arms—is also unlikely. Because survival did not differ significantly between the combination arms (*p* = 0.2), survivor bias is likely to act similarly in both and is unlikely to fully explain the higher cerebral SOD observed under protons + olaparib (+75.1%, q = 0.010), although this cannot be formally excluded. Within the proton arm, however, the catalase increase reached only nominal significance and did not survive FDR correction (+55.9%, *p* = 0.027, q = 0.27), while the SOD increase was not significant after day adjustment (+54.0%, *p* = 0.081), further constraining any mechanistic attribution. Reinforcing this picture, protons + olaparib also showed a directional reduction in cerebral protein carbonylation relative to photons + olaparib (−63.6%, *p* = 0.054, q = 0.270), which, although not surviving FDR correction, is directionally consistent with the higher cerebral SOD and warrants confirmation in future studies with larger sample sizes.

In the myocardium, the pre-specified day-adjusted analysis identified a more restricted set of modality-specific effects than unadjusted testing had suggested. Relative to photons alone, photons + olaparib showed a nominal increase in cardiac PARP1 activity (+145.3%, *p* = 0.025) and an FDR-significant reduction in the GSH/GSSG ratio (−26.1%, *p* = 0.002, q = 0.010), whereas the concurrent nominal increases in catalase, SOD, GPx, and GSH did not persist after adjustment for the day of sacrifice (all NS) and are therefore not emphasized. Proton irradiation alone selectively increased cardiac protein carbonylation (+223.5%, *p* = 0.021, q = 0.090) and GPx (+204.7%, *p* = 0.027, q = 0.090), although neither increase survived FDR correction; if confirmed, this would suggest that protons per se induce oxidative protein damage in the myocardium. The absence of a further carbonylation increase under protons + olaparib may reflect an adaptive ceiling effect rather than cardioprotection, although this was not tested. Critically, direct inter-arm comparison revealed no statistically significant difference in any cardiac biomarker between the combination arms (all NS), and conclusions regarding a differential cardiac biomarker profile should therefore be considered exploratory, pending dedicated studies with serial cardiac endpoints.

The liver stands apart from the eight other compartments, and this discordance is in itself informative: total hepatic protein content differed markedly between the two combination arms, whereas it was comparable between arms in every other tissue. Such an increase, observed here in the proton arms only, is not without precedent after whole-body irradiation. Hepatic protein biosynthesis in mice is unaffected below 6 Gy but rises with dose beyond that threshold, reaching 70–75% above control at the highest dose tested, an effect attributed to an expanded ribosomal population and a larger intracellular precursor pool [58]. Whole-body irradiation likewise induces the acute-phase protein response, which peaks between days 3 and 7 after exposure [59], which is the very window in which our combination arms were sampled (median day 8). Both mechanisms, however, are effects of irradiation itself and cannot account for a difference between two arms exposed to the same dose. PARP inhibition provides a mechanism that does distinguish them: by halting poly-ADP-ribose synthesis, it spares the NAD^+^ pool and preserves the energetic capacity required for protein synthesis [60] so that a modality-dependent difference in the extent of PARP-1 inhibition might plausibly translate into a difference in hepatic protein content. This difference carries a direct methodological consequence. The hepatic biomarkers are the only ones in the present dataset whose interpretation depends on the choice of denominator, and the apparent decreases observed after protons + olaparib cannot therefore be taken as evidence of reduced oxidative damage. The GSH/GSSG ratio, mathematically unaffected by protein normalization, remains the only hepatic measure supporting a genuine difference in redox balance between the two modalities. That an organ-specific divergence of this magnitude should arise in the liver alone—in a design where all nine compartments were sampled from the same animals under identical dosimetry—is precisely the signal this screen was intended to detect. It identifies the liver as the compartment warranting dedicated investigation, employing protein-independent normalization, direct measurement of hepatic protein content and tissue mass, and conventional hepatotoxicity endpoints.

Intestinal GPx was higher under protons + olaparib than under photons + olaparib (+449.9%, *p* = 0.014, q = 0.047), together with lower GSSG and a higher GSH/GSSG ratio (both q < 0.01). One possible, untested explanation is a compensatory antioxidant response in crypt cells facing proton-induced clustered DSBs when PARP inhibition impairs HR repair [15,16], in a context where SOD/catalase induction alone is insufficient to maintain redox homeostasis [61,62]. No DNA damage, crypt, or histological data were collected, and the functional significance of these differences must be tested in dedicated studies.

Thoracic skin showed high baseline PARP activity in control animals, which may reflect the constitutive role of PARP-1 in DNA repair in skin cells [63]. Relative to photons alone, photons + olaparib showed directional attenuation of cutaneous oxidative markers (LPO −42.7%, carbonylation −56.0%) that reached nominal significance on unadjusted testing but not after day adjustment (*p* = 0.103 and *p* = 0.058, respectively), with only SOD reaching nominal ANCOVA significance (−41.7%, *p* = 0.038); none survived FDR correction. In contrast, protons + olaparib showed a divergent cutaneous oxidative pattern on direct inter-arm comparison, characterized by higher protein carbonylation (+142.9%, *p* < 0.001, q = 0.002) and a lower GSH/GSSG ratio (−77.6%, *p* = 0.008, q = 0.036), both surviving FDR correction, together with near-complete GSH depletion (−95.7%, *p* = 0.023, q = 0.083, nominal only); directionally concordant within-modality changes versus protons alone (LPO +71.5%, *p* = 0.029; SOD −61.3%, *p* = 0.018) did not survive FDR correction. This inter-modality comparison should, however, be interpreted with particular caution. A residual air gap between the bolus and the skin surface in the photon arm may have introduced relative superficial skin underdosing despite TLD-verified dosimetry (±5%, IAEA TRS-398), although planned skin doses were comparable between arms (102.5% with photons and 97.2% with protons; Supplementary Table S1). Such underdosing would be expected to reduce oxidative damage in the photon arm specifically and could therefore contribute to the cutaneous differences between arms, confounding direct inter-modality comparison independently of any olaparib-specific effect. Given this dosimetric uncertainty, and in the absence of any histological skin assessment or clinical dermatitis scoring, the near-complete cutaneous GSH depletion observed under protons + olaparib—a finding of potential interest given GSH’s established role in keratinocyte survival, wound healing, and radiation dermatitis in preclinical models [44,45]—should be regarded as a hypothesis-generating signal rather than a clinically actionable finding, and does not on its own support a recommendation for prospective dermatitis monitoring.

Pulmonary biomarkers did not differ significantly between the two combination arms (all NS; protons + olaparib vs. photons + olaparib), and adding olaparib did not significantly modify any pulmonary marker within either modality. Relative to control, both irradiation modalities were associated with lower catalase activity (protons −48.8%, *p* = 0.005, q = 0.009; photons −34.2%, *p* = 0.035, nominal only, q = 0.079) and with a marked reduction in GSH (photons −69.6%, protons −78.8%, both q < 0.001). These changes therefore appear to be driven mainly by irradiation rather than by olaparib. They cannot be compared directly with earlier work combining olaparib with thoracic irradiation in mice, which reported an increased therapeutic index in a tumour-bearing model without measuring pulmonary oxidative markers [37]. Within the proton arm, lipid peroxidation was higher under protons + olaparib than under protons alone (+57.0%, *p* = 0.029, day-adjusted), but this difference did not survive FDR correction (q = 0.261) and should be considered a trend only (Supplementary Table S9). Finally, given the small sample size, the absence of a detectable inter-arm difference should be interpreted with caution.

Olaparib introduced modality-specific perturbations in inflammatory signalling. Most notably, a striking plasma TGF-β1 restoration was observed under protons + olaparib versus protons alone (+521.2%, *p* = 0.024, day-adjusted; q = 0.07), which was absent under photons + olaparib. This differential response could reflect a PARP1–Smad3 signalling interaction specifically engaged in the context of proton-induced DNA damage [64], although this interpretation remains hypothetical in the absence of phospho-Smad3 or TGF-β pathway gene-expression data. Given the established role of TGF-β1 as a central mediator of radiation-induced fibrosis [65,66], this exploratory finding may warrant the inclusion of TGF-β1 as an endpoint in future preclinical proton–PARPi studies, pending confirmation in dedicated mechanistic studies.

These findings should be interpreted in light of several limitations. First, the model is near-lethal whole-body irradiation producing acute radiation syndrome, not localized, fractionated proton radiotherapy. Only oxidative, inflammatory, and PARP-activity biomarkers were measured; no histological, hematological, clinical-chemistry, or functional endpoint was obtained. The data therefore cannot establish normal-tissue protection, comparative clinical toxicity, or a therapeutic window for proton radiotherapy. Second, the principal pre-specified comparison of this exploratory study was the head-to-head contrast of the two combination arms (protons + olaparib versus photons + olaparib), sampled at a median day 8 after D0. Survival did not differ significantly between these arms (log-rank *p* = 0.2, q = 0.33), but a non-significant test does not establish equivalence. Control versus olaparib alone (both sacrificed electively at day 18) was the only time-matched secondary comparison. Contrasts between arms sampled at different times (irradiation-only versus combination and irradiated versus unirradiated) are inherently confounded by sampling time; they are reported as exploratory and adjusted for the day of sacrifice (see statistical analysis). This adjustment limits, but cannot eliminate, the risk that differences in time to endpoint are misinterpreted as modality- or drug-specific effects.

The 7.5 Gy whole-body irradiation dose is supratherapeutic relative to clinical practice and was associated with high lethality in combination groups (>90%), precluding full biochemical characterization in survivors. This dose was deliberately selected based on prior work from our group [26] to generate a robust and detectable oxidative signal within an exploratory framework; future investigations should employ fractionated or sub-lethal doses (2–4 Gy) to better approximate clinical conditions and improve animal welfare. Moreover, single-dose whole-body irradiation produces systemic acute radiation syndrome, with concurrent hematopoietic and gastrointestinal injury, whereas normal-tissue exposure during clinical proton radiotherapy is localized, spatially heterogeneous, and generally fractionated; the present findings therefore cannot be extended to normal-tissue toxicity during clinical proton radiotherapy. No combination-specific dose-finding step preceded the study: 7.5 Gy was retained because modality-dependent differences were expected to be smaller than the overall effect of irradiation, and a staged escalation, although preferable and possibly able to identify a lower effective dose, would not have ensured detectable responses across all compartments.

Interpretation of the hepatic data is constrained in two respects. First, hepatic biomarkers are expressed per mg of total protein, and total protein content differed markedly between the two combination arms; the apparent decreases observed after protons + olaparib may therefore reflect this difference in denominator rather than a genuine reduction in oxidative damage. Only the GSH/GSSG ratio, which is invariant to this normalization, is unaffected by this limitation. Second, hepatotoxicity was not formally assessed, as AST, ALT, bilirubin, histopathology, and fibrosis markers were not measured, so that none of the oxidative changes reported here can be equated with hepatoprotection or its absence.

Olaparib plasma levels were not quantified; however, the 50 mg/kg oral regimen has been validated in murine preclinical models with documented target engagement and PARP inhibition at this dose [37,38], supporting the biological relevance of the pharmacological intervention.

The single terminal sampling per animal, while appropriate for an exploratory hypothesis-generating design, precludes assessment of temporal dynamics, and the heterogeneous sacrifice timing across groups introduces potential confounding. Fixed sampling times could not, however, have been imposed: they would have required either sacrificing animals before they reached their humane endpoint or maintaining them beyond it, with the latter being incompatible with EU Directive 2010/63/EU and with the humane endpoints set out in the project authorization. Because early mortality was comparable between the two combination arms, survivor bias would be expected to act broadly symmetrically on both, and is therefore unlikely to fully explain the directional divergence observed in the principal comparison. This argument, however, does not rule out a contribution of survivor bias to the absolute biomarker values observed within either arm, nor does it extend to the four remaining, non-synchronous comparisons, which are consequently reported as exploratory (rank-based ANCOVA adjusted for day of sacrifice, Supplementary Tables S4–S11) rather than as confirmatory findings. Individual biomarker values are displayed against the actual day of sacrifice to document the relationship with sampling time (Supplementary Figure S2). Because sacrifice days differed between arms by design (humane endpoint), this descriptive check cannot formally validate the day adjustment or exclude non-monotonic kinetics, and the day-adjusted comparisons are therefore considered exploratory. Longitudinal sampling will be needed in confirmatory studies to characterize kinetics and to disentangle survivor-bias effects from modality- or drug-specific dynamics.

Finally, clinical monitoring was performed five days per week and no group-level stopping rule was predefined; daily monitoring and a predefined group-level stopping rule would be incorporated into any future study using this regimen.

Across the compartments examined, radiation modality was associated with differences in the tissue oxidative response to olaparib. The direction of these differences was not uniform: intestinal, erythrocyte, and cerebral markers were higher under the proton combination (intestinal GSSG lower), whereas the cutaneous GSH/GSSG ratio was lower and protein carbonylation was higher. The hepatic differences cannot be attributed to either modality because of the protein-normalization confounder. These signals are exploratory observations and they identify compartments for targeted investigation rather than establishing a modality-dependent therapeutic window. Collectively, these results suggest that the oxidative response to PARP inhibitor–radiotherapy combinations may be modality-dependent in this model and should not be directly extrapolated between photon and proton treatments; whether organ-specific, modality-adapted monitoring strategies are warranted can only be determined in specifically designed studies.

## 5. Conclusions

In this exploratory, single-time-point, tumour-free preclinical model using a near-lethal whole-body dose—a design chosen to compare tissue redox biomarkers between radiation modalities independently of tumour-related confounders—this study enabled a comparative evaluation of PARP inhibition across multiple organs and tissue compartments simultaneously, within the same animals and under identical systemic conditions, an assessment that separate single-organ studies could not provide. Within these limits, the two combinations were associated with different tissue oxidative profiles, and the screen identifies the compartments in which these modality-associated differences are largest. In the principal inter-arm analysis, 18 of 74 biomarkers differed after FDR correction. Seven were hepatic values expressed per mg of total protein and were considered non-interpretable because total hepatic protein itself differed markedly between the two arms. The remaining 11 differences were as follows under protons + olaparib relative to photons + olaparib: higher hepatic total protein and GSH/GSSG ratio, higher cerebral SOD, higher intestinal GPx and GSH/GSSG ratio with lower GSSG, higher erythrocyte GSH, GSSG, and SOD, and, in the skin, a lower GSH/GSSG ratio with higher protein carbonylation. Inflammatory signalling, assessed through TGF-β1 and a 13-plex cytokine panel, showed no FDR-significant difference between the two combination arms; in the exploratory within-modality analysis, adding olaparib to protons, but not to photons, was associated with a higher plasma TGF-β1 relative to irradiation alone, a nominal signal that did not survive FDR correction.

Together, these oxidative stress and inflammatory signals are hypothesis-generating. They do not, on their own, establish a difference in toxicity between the two modalities, and the cutaneous differences, which went in the opposite direction to those in the other compartments, would require dedicated investigation. These exploratory findings—obtained in a design largely unprecedented in the literature for combining whole-body photon–proton comparison, PARP inhibition, and oxidative/inflammatory biomarkers—underscore the need for confirmatory studies in tumour-bearing models, using localized irradiation, combination-specific dose-finding, sub-lethal or fractionated dosing, histological and clinical-chemistry endpoints, and longitudinal sampling, before any translational or clinical inference can be drawn.

## Data Availability

Summary data supporting the findings of this study are presented in the supplementary tables. Individual animal-level datasets are available from the corresponding author upon reasonable request.

## Supplementary Materials

The following supporting information can be downloaded at: https://www.mdpi.com/article/10.3390/antiox15101365/s1, The following supporting information can be downloaded at the journal website. Table S1: Absorbed dose to each studied compartment under photon and proton whole-body irradiation; Figure S1: Timing of biological sampling across treatment arms; Table S2: Biomarkers assessed (biological category, technique, unit, tissues); Figure S2: Individual biomarker values versus actual day after irradiation for FDR-significant exploratory findings; Figure S3: Kaplan–Meier survival curves restricted to the 54 animals retained for biomarker analysis; Table S3: Multi-organ biomarker profile, control versus olaparib monotherapy; Table S4: Cerebral biomarker responses; Figure S4: Cerebral biomarker profiles across the six treatment groups; Tables S5–S10: Cardiac, hepatic, intestinal, cutaneous, pulmonary, and systemic biomarker responses to irradiation alone and in combination with olaparib; Table S11: Summary of day-adjusted (ANCOVA) comparisons across all tissue compartments; Figure S5: Standardized effect sizes for biomarkers differing between the two combination arms.
