# Supplementary material for: Photon Versus Proton Whole-Body Irradiation Combined with Olaparib in C57BL/6 Mice: An Exploratory Study of Oxidative Stress, Inflammatory, and DNA Damage Response Biomarkers
Source: Antioxidants (Basel). 2026 Oct 21;15(10):1365. doi: 10.3390/antiox15101365 (PMC13701196; doi:10.3390/antiox15101365)
Supplement: Supplementary file 1 [file antioxidants-15-01365-s001.zip › antioxidants-4545609-supplementary.pdf]

Supplementary Table S1. Absorbed dose to each studied compartment under photon and proton whole-body irradiation.

Structure	Photons		Protons	
	**D50% (Gy) mean ± SD**	**% of prescribed**	**D50% (Gy) mean ± SD**	**% of prescribed**
Body	7.65 ± 0.03	102.0	7.40 ± 0.01	98.6
Bone	7.54 ± 0.05	100.5	7.40 ± 0.01	98.6
Liver	7.58 ± 0.05	101.1	7.50 ± 0.00	100.0
Brain	7.62 ± 0.03	101.6	7.56 ± 0.01	100.8
Heart	7.62 ± 0.05	101.6	7.56 ± 0.01	100.8
Peritoneal cavity	7.66 ± 0.04	102.1	7.43 ± 0.01	99.0
Skin	7.69 ± 0.03	102.5	7.29 ± 0.02	97.2
Lungs	7.81 ± 0.05	104.1	7.50 ± 0.01	100.0

*Note***.** Doses were derived from treatment-planning dose–volume histograms for a 7.5 Gy whole-body prescription and are reported as the median absorbed dose D50% (mean ± SD across the three animals) and as a percentage of the prescribed dose. Both modalities delivered close to the prescribed dose in every compartment (photons 100–104 %, protons 97–101 %), and the between-animal reproducibility was high (SD ≤ 0.05 Gy). These small and comparable deviations suggest that the observed differences in biomarker responses between arms are unlikely to be explained solely by differences in absorbed dose. The largest proton deviation was in skin (7.29 ± 0.02 Gy, 97 %), reflecting the lateral penumbra of the scanned field at the body surface. *Abbreviations:* D50%, median dose (dose covering 50 % of the structure volume); SD, standard deviation.

Supplementary Figure S1. Timing of biological sampling across treatment arms

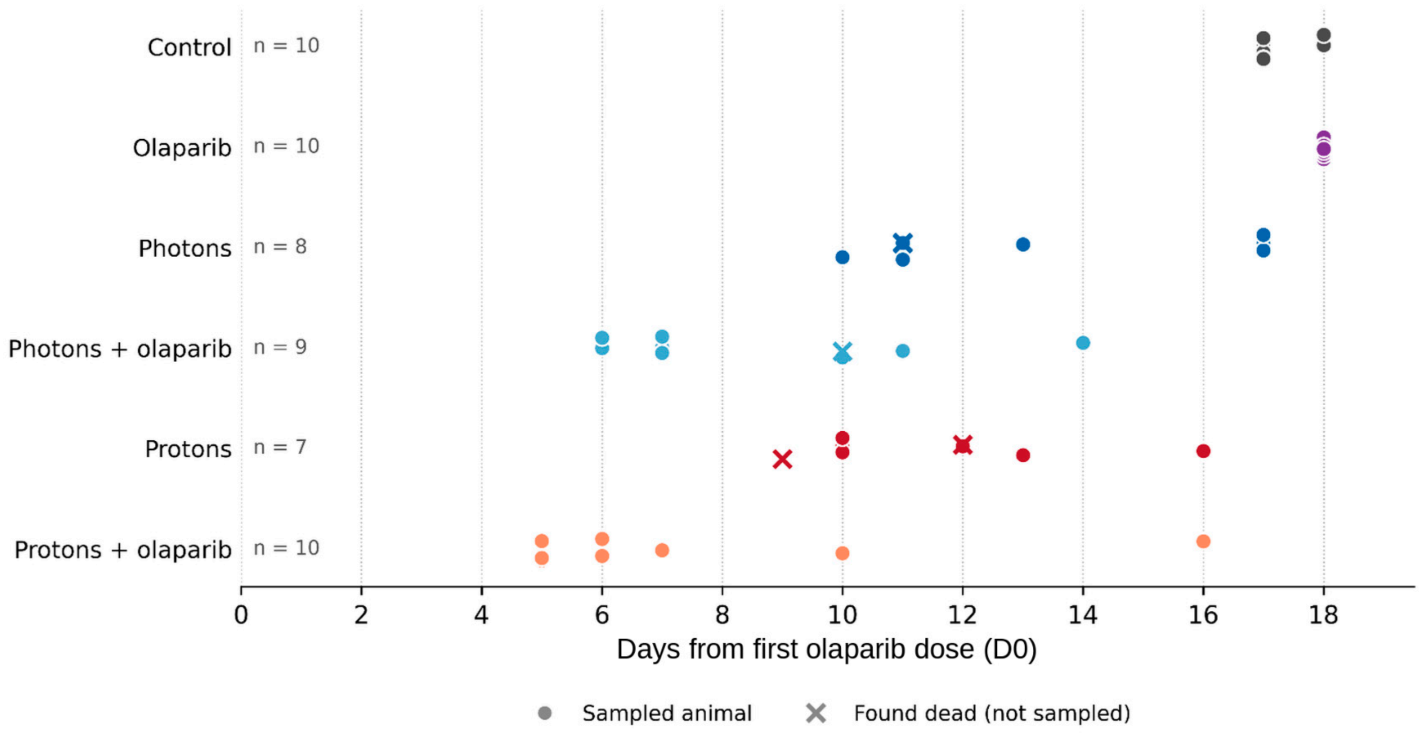

Note. Each symbol represents the sampling day of one individual mouse, expressed in days from the first olaparib dose (D0; whole-body irradiation delivered at D1), grouped by treatment arm; symbols sampled on the same day may appear superimposed. Animals in the irradiated arms were sampled at their predefined humane endpoint (>20 % body-weight loss or signs of severe distress), whereas control and olaparib-alone animals underwent scheduled sacrifice at day 18. Crosses indicate the six animals found dead before sampling (photons, n = 2; photons + olaparib, n = 1; protons, n = 3); they could not be sampled and are excluded from the biomarker analyses, but are retained as observed deaths in the survival analysis (Figure 2, n = 60). Effective biomarker sample sizes were n = 10 (control), 10 (olaparib), 8 (photons), 9 (photons + olaparib), 7 (protons) and 10 (protons + olaparib). Median time to biomarker sampling in the 54 animals analysed was 18 days in the unirradiated arms, 16 days for photons and 12 days for protons in the irradiation-only arms, and 8 days in both combination arms. Within each arm, animals were sampled at individual, endpoint-driven times. The combination arms were designated as the principal comparison because their sampling times were the closest; the corresponding Wilcoxon analysis is not adjusted for the individual day of sacrifice. This figure shows the spread of sampling times, which should be considered when interpreting the between-arm biomarker comparisons.

Supplementary Table S2. Biomarkers assessed: biological category, analytical technique, unit of measurement, and tissues analysed. All measurements were performed in duplicate.

	Biomarker	Biological category	Technique	Unit	Tissues / compartments
Total protein content					
1	**Total protein content**	General / structural	Bradford assay	*mg/mL (plasma) or mg/g tissue*	Plasma, Erythrocytes, Lymphocytes, Brain, Heart, Liver, Intestine, Skin/thorax, Lung
DNA oxidative damage					
2	**8-OXO-dG**	DNA oxidative damage	ELISA	*ng/mg protein*	Plasma, Lymphocytes
Protein oxidation					
3	**Protein carbonylation**	Protein oxidation	DNPH colorimetry	*nmol/mg protein*	Erythrocytes, Lymphocytes, Brain, Heart, Liver, Intestine, Skin/thorax, Lung
Antioxidant enzymes					
4	**Catalase**	Enzymatic antioxidant	Spectrophotometry	*nmol/min/mg protein*	Erythrocytes, Brain, Heart, Liver, Intestine, Skin/thorax, Lung
5	**GPx (glutathione peroxidase)**	Enzymatic antioxidant	Spectrophotometry	*IU/mg protein*	Erythrocytes, Brain, Heart, Liver, Intestine, Skin/thorax, Lung
6	**SOD (superoxide dismutase)**	Enzymatic antioxidant	Spectrophotometry	*U/mg protein*	Erythrocytes, Brain, Heart, Liver, Intestine, Skin/thorax, Lung
Glutathione status					
7	**GSH (reduced glutathione)**	Redox status	Fluorimetry / DTNB	*µM/mg protein*	Erythrocytes, Brain, Heart, Liver, Intestine, Skin/thorax, Lung
8	**GSSG (oxidized glutathione)**	Redox status	Fluorimetry / DTNB	*µM/mg protein*	Erythrocytes, Brain, Heart, Liver, Intestine, Skin/thorax, Lung
9	**GSH/GSSG ratio**	Redox status	Calculated from GSH & GSSG	*Ratio (dimensionless)*	Brain, Heart, Liver, Intestine, Skin/thorax, Lung
Lipid peroxidation					
10	**LPO / MDA (malondialdehyde)**	Lipid oxidation	TBARS assay	*nmol/mg protein*	Erythrocytes, lymphocytes, Brain, Heart, Liver, Intestine, Skin/thorax, Lung
DNA repair					
11	**PARP activity**	DNA repair	Colorimetric / ELISA	*nmol NAM/h/mg protein*	Lymphocytes, Brain, Heart, Liver, Intestine, Skin/thorax
Fibrosis marker					
12	**TGF-β1**	Fibrosis / inflammation	ELISA	*pg/mL*	Plasma
Systemic inflammation — multiplex immunoassay (plasma)					
13	**IL-23**	Pro-inflammatory	Multiplex immunoassay	*pg/mL*	Plasma
14	**IL-1α**	Pro-inflammatory	Multiplex immunoassay	*pg/mL*	Plasma
15	**IFN-γ**	Pro-inflammatory	Multiplex immunoassay	*pg/mL*	Plasma
16	**TNF-α**	Pro-inflammatory	Multiplex immunoassay	*pg/mL*	Plasma

	Biomarker	Biological category	Technique	Unit	Tissues / compartments
17	**MCP-1**	Chemokine	Multiplex immunoassay	*pg/mL*	Plasma
18	**IL-12p70**	Pro-inflammatory	Multiplex immunoassay	*pg/mL*	Plasma
19	**IL-1β**	Pro-inflammatory	Multiplex immunoassay	*pg/mL*	Plasma
20	**IL-10**	Anti-inflammatory	Multiplex immunoassay	*pg/mL*	Plasma
21	**IL-6**	Pro-inflammatory	Multiplex immunoassay	*pg/mL*	Plasma
22	**IL-27**	Immunomodulatory	Multiplex immunoassay	*pg/mL*	Plasma
23	**IL-17A**	Pro-inflammatory	Multiplex immunoassay	*pg/mL*	Plasma
24	**IFN-β**	Antiviral / inflammatory	Multiplex immunoassay	*pg/mL*	Plasma
25	**GM-CSF**	Haematopoietic	Multiplex immunoassay	*pg/mL*	Plasma

Note. Abbreviations: DNPH, 2,4-dinitrophenylhydrazine; DTNB, 5,5'-dithio-bis-(2-nitrobenzoic acid); GM-CSF, granulocyte-macrophage colony-stimulating factor; GPx, glutathione peroxidase; GSH, reduced glutathione; GSSG, oxidized glutathione; IFN, interferon; IL, interleukin; LPO, lipid peroxidation; MCP-1, monocyte chemoattractant protein-1; MDA, malondialdehyde; NAM, nicotinamide; PARP, poly(ADP-ribose) polymerase; SOD, superoxide dismutase; TBARS, thiobarbituric acid reactive substances; TGF-β1, transforming growth factor-β1; TNF-α, tumor necrosis factor-α; 8-OXO-dG, 8-oxo-7,8-dihydro-2'-deoxyguanosine.

Supplementary Figure S2. Individual biomarker values versus day after D0

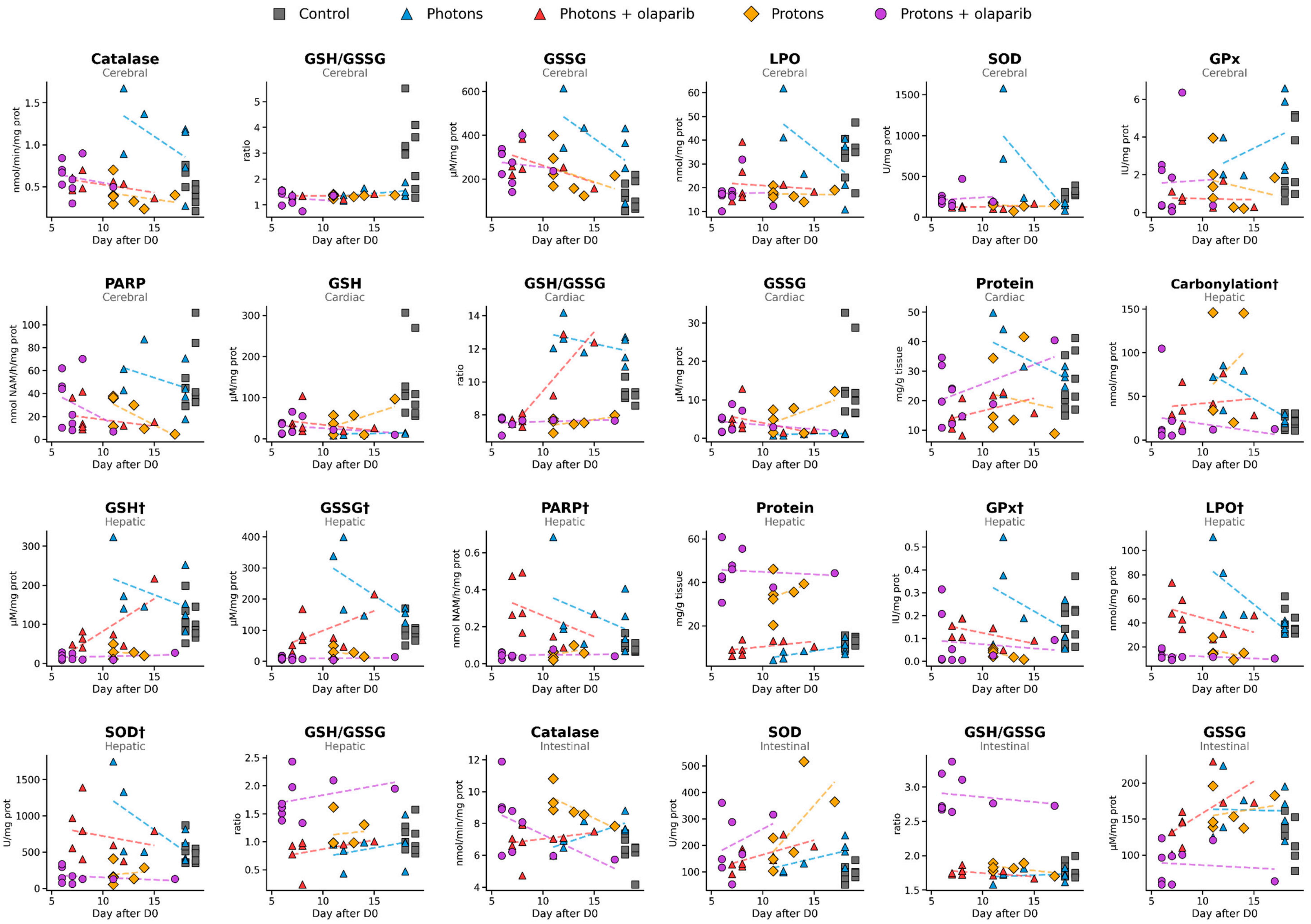

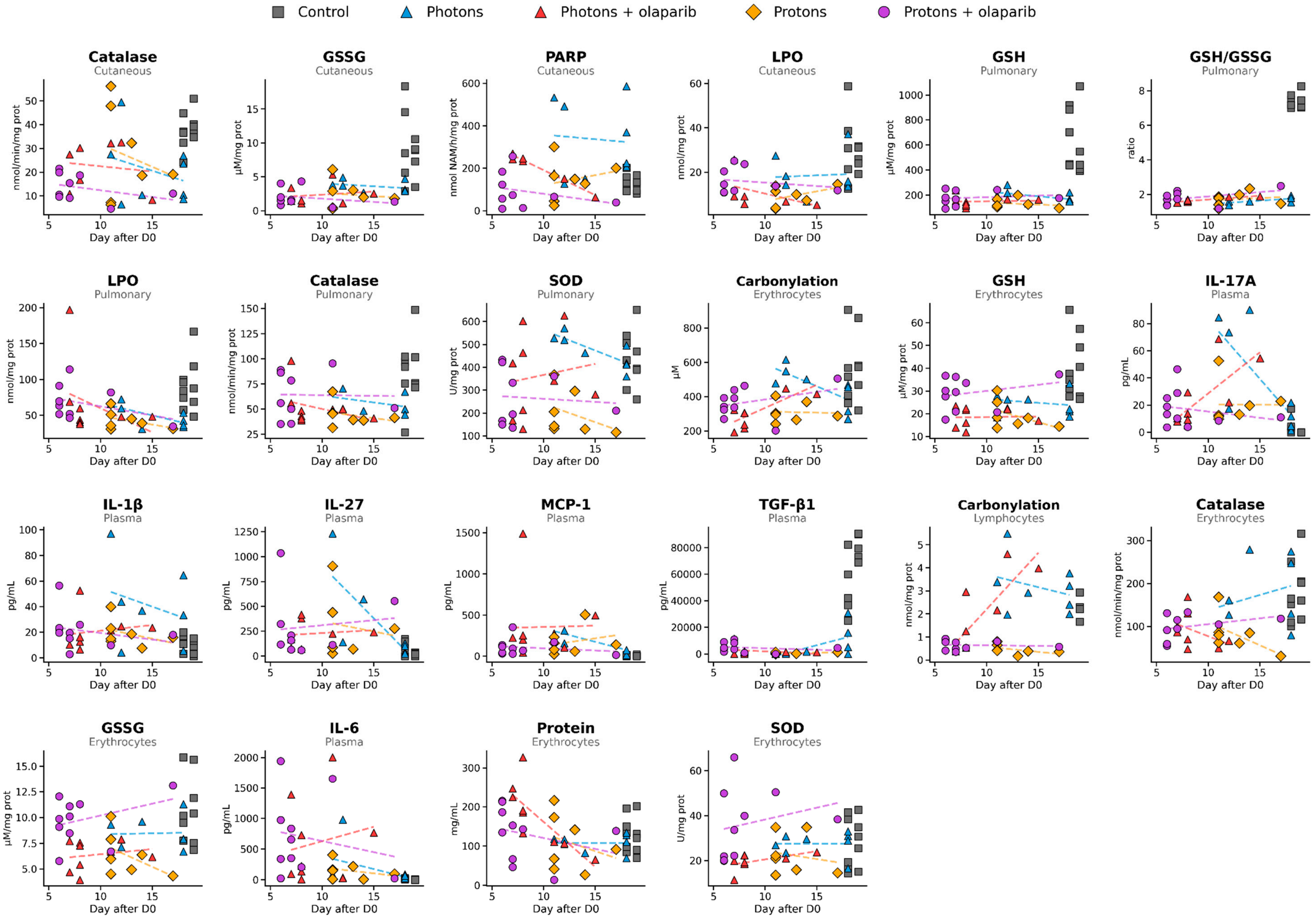

Note: The 46 biomarkers of Supplementary Table S11 (q_ANCOVA < 0.05 in at least one of the four day-adjusted comparisons) are shown in the order of that table. Each point is one animal at its actual day of sacrifice after D0 (D0 = first olaparib administration); animals found dead before sacrifice were excluded. Dashed lines are group-specific linear trends of the irradiated arms, shown as a visual aid, not a formal test. † Per mg of total hepatic protein, which differed markedly between groups (q < 0.001); not interpretable, shown for transparency. *Abbreviations: ANCOVA, analysis of covariance; GPx, glutathione peroxidase; GSH, reduced glutathione; GSSG, oxidized glutathione; IL, interleukin; LPO, lipid peroxidation; MCP-1, monocyte chemoattractant protein-1; PARP, poly(ADP-ribose) polymerase; SOD, superoxide dismutase; TGF-β1, transforming growth factor-β1.*

Supplementary Figure S3. Kaplan–Meier survival curves restricted to the 54 animals retained for biomarker analysis

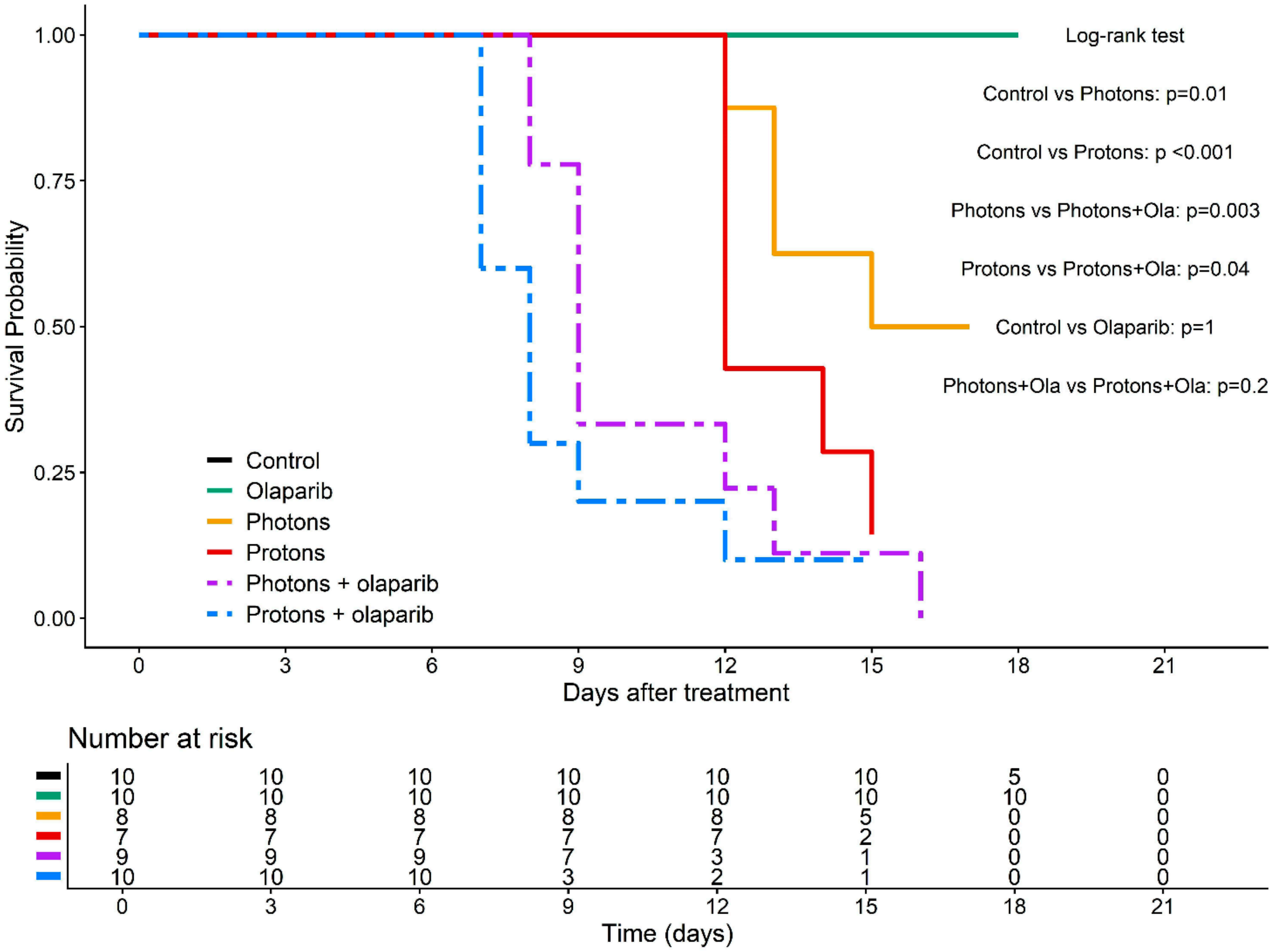

Note: Observation period: 18 days. Group sizes were n = 10 (control), 10 (olaparib), 8 (photons), 7 (protons), 9 (photons + olaparib) and 10 (protons + olaparib); the number at risk is shown below the plot. Compared with the main analysis (Figure 2, n = 60), this cohort excludes the six animals found dead and not sampled for biomarkers (photons, n = 2; photons + olaparib, n = 1; protons, n = 3). Median survival in this cohort was 15 and 12 days after photon and proton irradiation alone, and 9 and 8 days when combined with olaparib. Pairwise log-rank (Mantel–Cox) p-values are displayed on the plot; Benjamini–Hochberg q-values, applied across the six pre-specified pairwise comparisons, are given here: control vs. olaparib, p = 1 (q = 1); control vs. photons, p = 0.01 (q = 0.025); control vs. protons, p < 0.001 (q < 0.001); photons vs. photons + olaparib, p = 0.003 (q = 0.009); protons vs. protons + olaparib, p = 0.04 (q = 0.06); photons + olaparib vs. protons + olaparib, p = 0.2 (q = 0.24).

Supplementary Table S3. Multi-organ biomarker profile: Control versus Olaparib monotherapy (50 mg/kg/day).

Biomarker	Control Mean (SD)	Olaparib Mean (SD)	Δ%	Cohen's d	p	FDR q
**Liver**						
**Protein**	**12.5 (2.06)**	**19.1 (5.18)**	**+52.4%**	**−1.67**	**<0.001*** **	**0.022**
Carbonylation	20.5 (8.27)	20.0 (9.76)	−2.6%	0.06	0.971	0.993
Catalase	374 (402)	193 (99.6)	−48.3%	0.62	0.393	0.749
GPx	0.172 (0.0991)	0.171 (0.120)	−0.5%	0.01	0.912	0.993
GSH	105 (43.8)	72.2 (39.0)	−31.0%	0.78	0.052	0.271
GSSG	95.9 (31.3)	100 (54.5)	+4.2%	−0.09	0.436	0.767
GSH/GSSG	1.08 (0.243)	0.847 (0.475)	−21.9%	0.63	0.052	0.271
LPO/MDA	40.1 (9.99)	31.0 (6.29)	−22.7%	1.09	0.019*	0.150
**PARP**	**0.0966 (0.0305)**	**0.0550 (0.0167)**	**−43.0%**	**1.69**	**0.002** **	**0.033**
SOD	497 (157)	344 (97.2)	−30.8%	1.17	0.009**	0.098
**Brain**						
Protein	2.41 (0.595)	2.46 (0.650)	+2.1%	−0.08	0.905	0.993
Carbonylation	25.4 (38.9)	29.5 (28.4)	+15.9%	−0.12	0.165	0.520
Catalase	0.511 (0.192)	0.533 (0.235)	+4.2%	−0.10	0.968	0.993
GPx	2.35 (1.81)	4.02 (3.37)	+71.0%	−0.61	0.156	0.520
GSH	336 (109)	322 (93.9)	−4.2%	0.14	0.905	0.993
GSSG	126 (59.5)	213 (70.8)	+68.9%	−1.33	0.019*	0.150
GSH/GSSG	3.08 (1.30)	1.79 (0.700)	−41.8%	1.23	0.032*	0.213
LPO/MDA	34.1 (8.70)	36.7 (6.29)	+7.7%	−0.35	0.661	0.908
PARP	51.4 (27.9)	40.4 (14.1)	−21.4%	0.51	0.400	0.749
**SOD**	**293 (45.9)**	**459 (138)**	**+56.7%**	**−1.58**	**0.002** **	**0.037**
**Heart**						
Protein	25.4 (9.33)	24.9 (8.49)	−2.1%	0.06	0.971	0.993
Carbonylation	280 (186)	216 (99.7)	−22.9%	0.43	0.796	0.987
Catalase	1.03 (0.837)	1.05 (0.833)	+1.0%	−0.01	0.971	0.993
GPx	0.161 (0.174)	0.117 (0.115)	−27.6%	0.30	0.971	0.993
GSH	130 (87.3)	103 (109)	−20.2%	0.27	0.190	0.540
GSSG	14.0 (9.21)	10.7 (12.4)	−23.3%	0.30	0.143	0.504
GSH/GSSG	9.21 (0.490)	11.7 (3.23)	+26.7%	−1.06	0.089	0.357
LPO/MDA	60.8 (47.3)	47.5 (29.1)	−21.8%	0.33	0.661	0.908
PARP	0.117 (0.0683)	0.0926 (0.0499)	−20.6%	0.40	0.529	0.812
SOD	112 (119)	64.1 (47.0)	−42.8%	0.53	0.436	0.767
**Lung**						
Protein	4.70 (1.53)	4.87 (1.30)	+3.5%	−0.12	0.971	0.993
Carbonylation	56.4 (22.9)	66.4 (26.7)	+17.7%	−0.40	0.280	0.632
Catalase	86.7 (31.0)	80.5 (31.1)	−7.2%	0.20	0.315	0.677
GPx	1.12 (0.724)	0.847 (0.462)	−24.1%	0.44	0.353	0.722
GSH	626 (250)	441 (281)	−29.6%	0.70	0.315	0.677
GSSG	85.0 (34.2)	90.1 (33.2)	+6.0%	−0.15	0.853	0.993
GSH/GSSG	7.38 (0.388)	5.17 (2.82)	−29.9%	1.10	0.052	0.271
LPO/MDA	90.2 (33.9)	75.1 (18.0)	−16.7%	0.56	0.248	0.605
SOD	445 (115)	378 (135)	−15.0%	0.53	0.353	0.722
**Intestine**						
Protein	8.08 (1.68)	9.50 (0.749)	+17.5%	−1.11	0.036*	0.226
Carbonylation	72.6 (28.7)	54.9 (34.5)	−24.3%	0.55	0.236	0.605
Catalase	6.26 (0.946)	6.46 (0.492)	+3.2%	−0.27	0.606	0.888
GPx	0.992 (0.608)	0.340 (0.157)	−65.7%	1.51	0.006**	0.073
GSH	227 (49.7)	185 (16.0)	−18.4%	1.16	0.027*	0.201
GSSG	126 (28.1)	103 (8.51)	−18.3%	1.14	0.059	0.289
GSH/GSSG	1.80 (0.115)	1.80 (0.0939)	−0.1%	0.02	0.963	0.993
LPO/MDA	61.5 (28.2)	56.7 (10.7)	−7.8%	0.23	0.541	0.812
PARP	2.04 (0.622)	2.50 (0.827)	+22.7%	−0.63	0.139	0.504
SOD	95.9 (27.8)	116 (73.9)	+21.3%	−0.36	0.743	0.976
**Thoracic skin**						
Protein	1.36 (0.418)	1.62 (0.539)	+19.0%	−0.54	0.529	0.812
Carbonylation	19.8 (11.2)	16.6 (8.70)	−16.2%	0.32	0.393	0.749
Catalase	37.7 (7.15)	21.7 (20.6)	−42.4%	1.04	0.063	0.292
GPx	5.34 (3.91)	3.20 (2.68)	−40.0%	0.64	0.190	0.540
GSH	10.4 (9.66)	5.14 (4.25)	−50.4%	0.70	0.218	0.580
GSSG	8.93 (4.75)	7.54 (3.49)	−15.5%	0.33	0.529	0.812
GSH/GSSG	1.35 (1.23)	0.716 (0.159)	−46.9%	0.72	0.190	0.540
LPO/MDA	29.4 (12.7)	27.2 (8.71)	−7.7%	0.21	0.796	0.987

PARP	129 (38.5)	102 (36.6)	−20.6%	0.71	0.166	0.520
SOD	58.0 (20.2)	48.8 (9.93)	−15.8%	0.58	0.393	0.749
**Lymphocytes**						
Protein	44.1 (15.8)	44.8 (10.1)	+1.6%	−0.06	0.945	0.993
Carbonylation	2.27 (0.520)	2.62 (1.09)	+15.2%	−0.35	0.539	0.812
LPO/MDA	3.98 (1.43)	3.01 (0.812)	−24.3%	0.96	0.142	0.504
PARP	1.78 (0.510)	2.15 (0.821)	+20.4%	−0.48	0.733	0.976
8-OXO-dG	17.3 (5.72)	16.6 (5.78)	−3.8%	0.11	0.839	0.993
**Erythrocytes**						
Protein	128 (44.9)	195 (63.6)	+53.0%	−1.23	0.015*	0.144
Carbonylation	556 (194)	411 (126)	−26.0%	0.89	0.123	0.47
**Catalase**	**178 (67.8)**	**81.9 (31.0)**	**−54.0%**	**1.82**	**<0.001*** **	**0.022**
**GPx**	**0.0635 (0.0417)**	**0.0166 (0.00923)**	**−73.8%**	**1.59**	**0.001** **	**0.029**
GSH	39.8 (13.6)	29.7 (8.86)	−25.3%	0.88	0.089	0.357
GSSG	10.3 (3.27)	9.26 (3.62)	−10.5%	0.32	0.280	0.632
LPO/MDA	236 (99.7)	163 (66.1)	−30.7 %	0.85	0.105	0.42
SOD	27.8 (11.2)	27.2 (8.36)	−2.2%	0.06	1.000	1.000
**Plasma**						
Protein	60.1 (15.7)	65.5 (15.7)	+8.9%	−0.34	0.796	0.987
8-OXO-dG	1.31 (0.678)	1.16 (0.560)	−11.9%	0.25	0.796	0.987
TGF-β	64,900 (23,200)	73,200 (35,500)	+12.9%	−0.28	0.529	0.812
IL-23	7.32 (12.7)	4.16 (6.60)	−43.1%	0.31	0.545	0.812
IL-1α	3.08 (2.48)	3.29 (4.61)	+6.8%	−0.06	0.436	0.767
IFN-γ	2.59 (3.47)	1.10 (1.27)	−57.4%	0.57	0.209	0.574
TNF-α	2.41 (2.07)	2.59 (3.24)	+7.5%	−0.07	0.630	0.908
MCP-1	9.81 (6.51)	11.4 (9.47)	+16.2%	−0.20	0.912	0.993
IL-12p70	1.27 (2.95)	0 (0)	−100.0%	0.61	0.078	0.343
IL-1β	10.8 (6.46)	31.1 (39.5)	+187.6%	−0.72	0.248	0.605
IL-10	29.5 (35.9)	19.3 (19.3)	−34.5%	0.35	0.705	0.955
IL-6	9.91 (10.1)	4.26 (4.88)	−57.1%	0.71	0.269	0.632
IL-27	48.4 (63.9)	112 (195)	+130.7%	−0.44	0.469	0.810
IL-17A	4.21 (7.14)	2.76 (2.87)	−34.6%	0.27	0.500	0.812
IFN-β	31.9 (36.1)	22.7 (16.4)	−28.7%	0.33	1.000	1.000
GM-CSF	2.09 (2.40)	1.43 (1.62)	−31.5%	0.32	0.646	0.908

Note. Values are group means with standard deviation (SD). Δ% is the percentage change of the olaparib group relative to control. Effect sizes are expressed as Cohen's d. p-values are from two-sided Wilcoxon rank-sum tests; significance: † p ≤ 0.05; * p < 0.05; ** p < 0.01; *** p < 0.001. The false discovery rate was controlled across all biomarkers using the Benjamini–Hochberg procedure; the FDR q column reports the adjusted p-value for every biomarker, with q < 0.05 considered significant after correction. Bold entries indicate values that remain statistically significant after Benjamini–Hochberg FDR correction (q < 0.05). Biomarkers are grouped by tissue; organs are ordered following their citation in the main text. *Abbreviations: GM-CSF, granulocyte–macrophage colony-stimulating factor; GPx, glutathione peroxidase; GSH, reduced glutathione; GSSG, oxidized glutathione; IFN, interferon; IL, interleukin; LPO/MDA, lipid peroxidation / malondialdehyde; PARP, poly(ADP-ribose) polymerase; SOD, superoxide dismutase; TGF-β, transforming growth factor-β; TNF-α, tumor necrosis factor-α.*

Supplementary Table S4. Cerebral biomarker responses to irradiation alone and in combination with olaparib.

**Biomarker**	**Photons vs. control**	**Protons vs. control**	**Photons vs. Photons+Olaparib**	**Protons vs. Protons+Olaparib**	**Protons+Olaparib vs. Photons+Olaparib**
**SOD**	NS	**-50.7%*** (d=3.57, p=<0.001, q=<0.001)**	NS	NS	**+75.1%** (d=1.28, p=0.001, q=0.010)**
**Catalase**	**+103.7%** (d=−1.59, p=0.003, q=0.015)**	NS	**-49.0%* (d=1.54, p=0.011, q=0.037)**	+55.9%* (d=−1.27, p=0.027, q=0.270)	NS
**GPx**	NS	NS	**-79.5%** (d=2.10, p=0.002, q=0.010)**	NS	NS
**GSH**	+55.6%* (d=−1.09, p=0.032, q=0.080)	NS	NS	NS	NS
**GSSG**	**+186.6%** (d=−2.03, p=0.001, q=0.010)**	+79.0%* (d=−1.31, p=0.021, q=0.053)	NS	NS	NS
**GSH/GSSG**	**-52.2%** (d=1.61, p=0.009, q=0.030)**	**-56.2%** (d=1.76, p=0.005, q=0.017)**	NS	NS	NS
**LPO/MDA**	NS	**-49.2%*** (d=2.49, p=<0.001, q=<0.001)**	-38.2%* (d=1.06, p=0.046, q=0.115)	NS	NS
**PARP1 activity**	NS	-53.9%* (d=1.20, p=0.036, q=0.072)	**-65.1%** (d=1.90, p=0.002, q=0.010)**	NS	NS
**Protein**	NS	NS	NS	NS	NS
**Carbonylation**	NS	NS	NS	NS	NS

Note. Values are percentage changes relative to the indicated comparator group, with Cohen's d, p-value and Benjamini–Hochberg FDR q-value. The four time-shifted comparisons (Photons vs. control; Protons vs. control; Photons vs. Photons+Olaparib; Protons vs. Protons+Olaparib) were analysed by rank-based ANCOVA adjusted for the day of sacrifice, and report the day-adjusted p and its FDR-adjusted q (q_ANCOVA). The main comparison, protons + olaparib vs. photons + olaparib (median sacrifice day 8 after D0), was tested with a two-sided Wilcoxon–Mann–Whitney rank-sum test. P-values and FDR q-values are unadjusted for each animal's day of sacrifice. NS: not significant after FDR (q ≥ 0.05). Significance: * p < 0.05; ** p < 0.01; *** p < 0.001. Bold entries indicate values that remain statistically significant after Benjamini–Hochberg FDR correction (q < 0.05; q_ANCOVA for the four ANCOVA comparisons and q for the Wilcoxon comparison). All measurements in cerebral tissue homogenates. *Abbreviations: GPx, glutathione peroxidase; GSH, reduced glutathione; GSSG, oxidized glutathione; LPO/MDA, lipid peroxidation / malondialdehyde; PARP1, poly(ADP-ribose) polymerase 1; SOD, superoxide dismutase.*

Supplementary Figure S4. Cerebral biomarker responses across the six treatment groups.

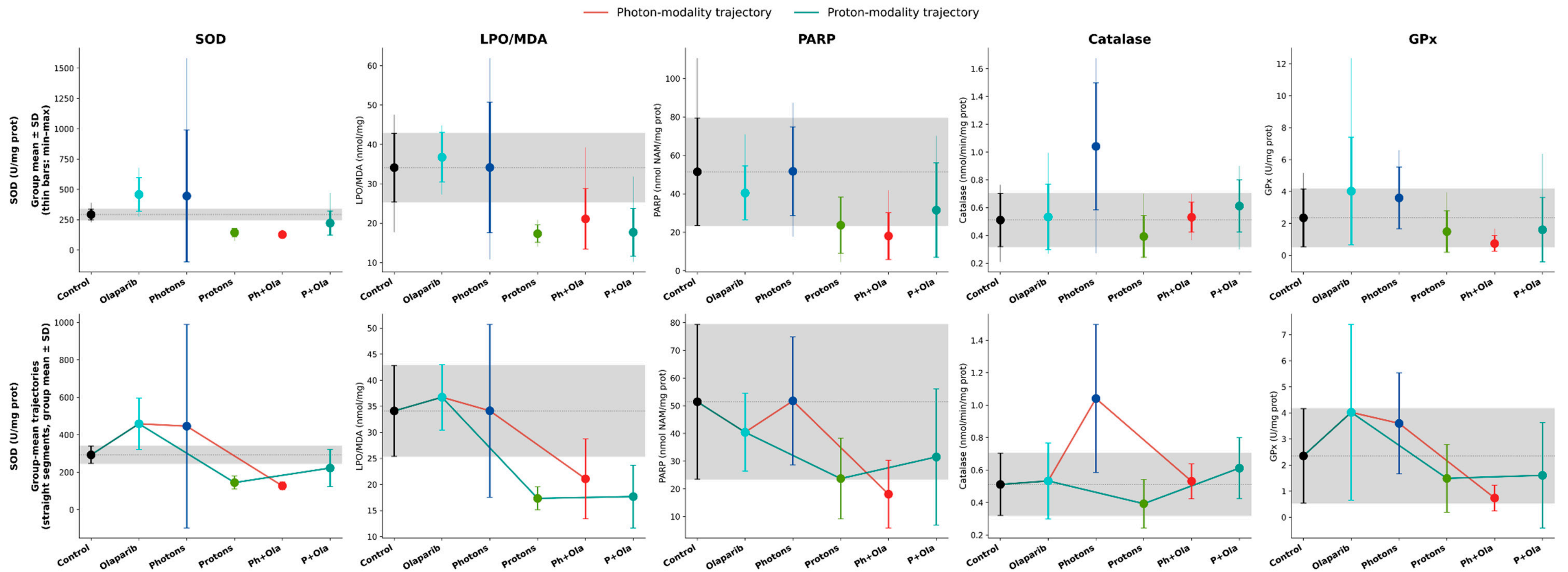

Note: Cerebral biomarker profiles (SOD, LPO/MDA, PARP, catalase, GPx) across the six treatment groups. Top row: group mean ± SD (thick error bars) with min–max range (thin bars). Bottom row: the same group means connected by straight line segments along each irradiation modality (photon-modality: Control → Olaparib → Photons → Ph+Ola; proton-modality: Control → Olaparib → Protons → P+Ola), shown purely to aid visual comparison across groups. Grey band: control mean ± 1 SD. This figure is descriptive only. Statistical comparisons (Wilcoxon and day-adjusted ANCOVA p/q-values) for these biomarkers are reported in Table 1 and Supplementary Table S4, and are not reproduced here. Line segments join observed group means only and do not represent an interpolated or modelled dose–response.

Supplementary Table S5. Cardiac biomarker responses to irradiation alone and in combination with olaparib.

Biomarker	Photons vs. control	Protons vs. control	Photons vs. Photons+Olaparib	Protons vs. Protons+Olaparib	Protons+Olaparib vs. Photons+Olaparib
**SOD**	NS	NS	NS	NS	NS
**Catalase**	NS	NS	NS	NS	NS
**GPx**	NS	+204.7%* (d=−1.41, p=0.027, q=0.090)	NS	NS	NS
**GSH**	**-89.8%** (d=1.77, p=0.003, q=0.010)**	-65.4%* (d=1.16, p=0.048, q=0.120)	NS	NS	NS
**GSSG**	**-92.2%** (d=1.86, p=0.002, q=0.010)**	NS	+288.8%* (d=−1.21, p=0.031, q=0.078)	NS	NS
**GSH/GSSG**	**+33.5%*** (d=−4.12, p=<0.001, q=<0.001)**	**-18.2%*** (d=3.73, p=<0.001, q=<0.001)**	**-26.1%** (d=1.83, p=0.002, q=0.010)**	NS	NS
**LPO/MDA**	NS	NS	NS	NS	NS
**PARP1 activity**	-52.4%* (d=1.16, p=0.032, q=0.080)	NS	+145.3%* (d=−1.25, p=0.025, q=0.078)	NS	NS
**Protein**	NS	NS	**-50.6%** (d=2.14, p=0.001, q=0.010)**	NS	NS
**Carbonylation**	NS	+223.5%* (d=−1.04, p=0.021, q=0.090)	NS	NS	NS

Note. Values are percentage changes relative to the indicated comparator group, with Cohen's d, p-value and Benjamini–Hochberg FDR q-value. The four time-shifted comparisons (Photons vs. control; Protons vs. control; Photons vs. Photons+Olaparib; Protons vs. Protons+Olaparib) were analysed by rank-based ANCOVA adjusted for the day of sacrifice, and report the day-adjusted p and its FDR-adjusted q (q_ANCOVA). The main comparison, protons + olaparib vs. photons + olaparib (median sacrifice day 8 after D0), was tested with a two-sided Wilcoxon–Mann–Whitney rank-sum test. P-values and FDR q-values are unadjusted for each animal's day of sacrifice. NS: not significant after FDR (q ≥ 0.05). Significance: * p < 0.05; ** p < 0.01; *** p < 0.001. Bold entries indicate values that remain statistically significant after Benjamini–Hochberg FDR correction (q < 0.05; q_ANCOVA for the four ANCOVA comparisons and q for the Wilcoxon comparison). All measurements in cardiac tissue homogenates. *Abbreviations: GPx, glutathione peroxidase; GSH, reduced glutathione; GSSG, oxidized glutathione; LPO/MDA, lipid peroxidation / malondialdehyde; PARP1, poly(ADP-ribose) polymerase 1; SOD, superoxide dismutase.*

Supplementary Table S6. Hepatic biomarker responses to irradiation alone and in combination with olaparib.

Biomarker	Photons vs. control	Protons vs. control	Photons vs. Photons+Olaparib	Protons vs. Protons+Olaparib	Protons+Olaparib vs. Photons+Olaparib
**SOD†**	+59.7%* (d=−0.86, p=0.043, q=0.061)	**-59.8%** (d=2.03, p=0.002, q=0.004)**	NS	NS	**-77.8%*** (d=−2.40, p=<0.001, q=<0.001)**
**Catalase†**	NS	NS	-78.8%* (d=1.11, p=0.028, q=0.093)	NS	**-87.5%*** (d=−1.74, p=<0.001, q=<0.001)**
**GPx†**	NS	**-81.7%** (d=1.75, p=0.006, q=0.010)**	NS	NS	NS
**GSH†**	**+66.3%* (d=−1.14, p=0.024, q=0.048)**	**-73.5%** (d=2.14, p=0.001, q=0.003)**	-57.4%* (d=1.44, p=0.016, q=0.090)	NS	**-77.1%*** (d=−1.36, p=<0.001, q=<0.001)**
**GSSG†**	**+118.1%** (d=−1.60, p=0.001, q=0.005)**	**-72.2%*** (d=2.60, p=<0.001, q=<0.001)**	-55.8%* (d=1.37, p=0.018, q=0.090)	**-63.1%** (d=1.69, p=0.009, q=0.045)**	**-89.3%*** (d=−1.85, p=<0.001, q=<0.001)**
**GSH/GSSG**	NS	NS	NS	**+55.2%** (d=−1.91, p=0.003, q=0.030)**	**+108.2%*** (d=2.89, p=<0.001, q=<0.001)**
**LPO/MDA†**	+35.6%* (d=−0.72, p=0.042, q=0.061)	**-60.4%*** (d=2.74, p=<0.001, q=<0.001)**	NS	NS	**-71.9%*** (d=−3.00, p=<0.001, q=<0.001)**
**PARP1 activity†**	**+165.5%* (d=−1.18, p=0.020, q=0.048)**	**-41.2%* (d=1.35, p=0.025, q=0.031)**	NS	NS	**-82.9%*** (d=−2.23, p=<0.001, q=<0.001)**
**Protein**	**-32.2%** (d=1.49, p=0.002, q=0.007)**	**+176.6%*** (d=−4.12, p=<0.001, q=<0.001)**	NS	+30.4%* (d=−1.20, p=0.050, q=0.150)	**+344.9%*** (d=5.11, p=<0.001, q=<0.001)**
**Carbonylation†**	**+138.9%** (d=−1.47, p=0.001, q=0.005)**	**+270.2%* (d=−1.53, p=0.017, q=0.024)**	NS	NS	**-48.7%* (d=−0.73, p=0.012, q=0.013)**

Note. Values are percentage changes relative to the indicated comparator group, with Cohen's d, p-value and Benjamini–Hochberg FDR q-value. The four time-shifted comparisons (Photons vs. control; Protons vs. control; Photons vs. Photons+Olaparib; Protons vs. Protons+Olaparib) were analysed by rank-based ANCOVA adjusted for the day of sacrifice, and report the day-adjusted p and its FDR-adjusted q (q_ANCOVA). The main comparison, protons + olaparib vs. photons + olaparib (median sacrifice day 8 after D0), was tested with a two-sided Wilcoxon–Mann–Whitney rank-sum test. P-values and FDR q-values are unadjusted for each animal's day of sacrifice. NS: not significant after FDR (q ≥ 0.05). Significance: * p < 0.05; ** p < 0.01; *** p < 0.001. Bold entries indicate values that remain statistically significant after Benjamini–Hochberg FDR correction (q < 0.05; q_ANCOVA for the four ANCOVA comparisons and q for the Wilcoxon comparison). All measurements in hepatic tissue homogenates. † Per mg of total hepatic protein, which differed markedly between groups (q < 0.001); not interpretable, shown for transparency. *Abbreviations: GPx, glutathione peroxidase; GSH, reduced glutathione; GSSG, oxidized glutathione; LPO/MDA, lipid peroxidation / malondialdehyde; PARP1, poly(ADP-ribose) polymerase 1; SOD, superoxide dismutase.*

Supplementary Table S7. Intestinal biomarker responses to irradiation alone and in combination with olaparib.

Biomarker	Photons vs. control	Protons vs. control	Photons vs. Photons+Olaparib	Protons vs. Protons+Olaparib	Protons+Olaparib vs. Photons+Olaparib
**SOD**	+58.8%** (d=−1.42, p=0.006, q=0.060)	**+166.7%** (d=−1.55, p=0.004, q=0.020)**	NS	NS	NS
**Catalase**	+18.3%* (d=−1.24, p=0.019, q=0.095)	**+44.0%*** (d=−2.84, p=<0.001, q=<0.001)**	NS	NS	NS
**GPx**	NS	NS	NS	NS	**+449.9%* (d=1.21, p=0.014, q=0.047)**
**GSH**	NS	+27.4%* (d=−1.36, p=0.034, q=0.100)	NS	NS	NS
**GSSG**	+28.8%* (d=−1.12, p=0.049, q=0.143)	+26.3%* (d=−1.24, p=0.050, q=0.100)	NS	**-45.1%*** (d=2.81, p=<0.001, q=<0.001)**	**-42.7%*** (d=−1.98, p=<0.001, q=0.003)**
**GSH/GSSG**	NS	NS	NS	**+58.3%*** (d=−4.87, p=<0.001, q=<0.001)**	**+64.1%*** (d=5.73, p=<0.001, q=<0.001)**
**LPO/MDA**	NS	NS	NS	NS	NS
**PARP1 activity**	NS	NS	NS	NS	NS
**Protein**	NS	-25.2%* (d=1.27, p=0.044, q=0.100)	NS	NS	NS
**Carbonylation**	NS	NS	-34.3%* (d=1.17, p=0.032, q=0.320)	NS	NS

Note. Values are percentage changes relative to the indicated comparator group, with Cohen's d, p-value and Benjamini–Hochberg FDR q-value. The four time-shifted comparisons (Photons vs. control; Protons vs. control; Photons vs. Photons+Olaparib; Protons vs. Protons+Olaparib) were analysed by rank-based ANCOVA adjusted for the day of sacrifice, and report the day-adjusted p and its FDR-adjusted q (q_ANCOVA). The main comparison, protons + olaparib vs. photons + olaparib (median sacrifice day 8 after D0), was tested with a two-sided Wilcoxon–Mann–Whitney rank-sum test. P-values and FDR q-values are unadjusted for each animal's day of sacrifice. NS: not significant after FDR (q ≥ 0.05). Significance: * p < 0.05; ** p < 0.01; *** p < 0.001. Bold entries indicate values that remain statistically significant after Benjamini–Hochberg FDR correction (q < 0.05; q_ANCOVA for the four ANCOVA comparisons and q for the Wilcoxon comparison). All measurements in intestinal tissue homogenates. *Abbreviations: GPx, glutathione peroxidase; GSH, reduced glutathione; GSSG, oxidized glutathione; LPO/MDA, lipid peroxidation / malondialdehyde; PARP1, poly(ADP-ribose) polymerase 1; SOD, superoxide dismutase.*

Supplementary Table S8. Cutaneous biomarker responses to irradiation alone and in combination with olaparib.

Biomarker	Photons vs. control	Protons vs. control	Photons vs. Photons+Olaparib	Protons vs. Protons+Olaparib	Protons+Olaparib vs. Photons+Olaparib
**SOD**	NS	NS	-41.7%* (d=1.11, p=0.038, q=0.165)	-61.3%* (d=1.40, p=0.018, q=0.116)	-49.1%* (d=−1.27, p=0.031, q=0.083)
**Catalase**	**-45.8%** (d=1.56, p=0.005, q=0.025)**	NS	NS	NS	NS
**GPx**	NS	-79.9%* (d=1.39, p=0.015, q=0.060)	NS	NS	NS
**GSH**	NS	-86.2%* (d=1.16, p=0.038, q=0.101)	NS	NS	-95.7%* (d=−1.22, p=0.023, q=0.083)
**GSSG**	**-59.8%** (d=1.48, p=0.008, q=0.027)**	NS	NS	NS	NS
**GSH/GSSG**	NS	NS	NS	NS	**-77.6%* (d=-1.52, p=0.008, q=0.036)**
**LPO/MDA**	NS	**-68.5%** (d=1.97, p=0.002, q=0.016)**	NS	+71.5%* (d=−1.28, p=0.029, q=0.116)	NS
**PARP1 activity**	**+161.2%** (d=−1.67, p=0.004, q=0.025)**	NS	NS	NS	-53.8%* (d=−1.36, p=0.042, q=0.084)
**Protein**	NS	NS	NS	NS	NS
**Carbonylation**	NS	NS	NS	NS	**+142.9%*** (d=0.97, p=<0.001, q=0.002)**

Note. Values are percentage changes relative to the indicated comparator group, with Cohen's d, p-value and Benjamini–Hochberg FDR q-value. The four time-shifted comparisons (Photons vs. control; Protons vs. control; Photons vs. Photons+Olaparib; Protons vs. Protons+Olaparib) were analysed by rank-based ANCOVA adjusted for the day of sacrifice, and report the day-adjusted p and its FDR-adjusted q (q_ANCOVA). The main comparison, protons + olaparib vs. photons + olaparib (median sacrifice day 8 after D0), was tested with a two-sided Wilcoxon–Mann–Whitney rank-sum test. P-values and FDR q-values are unadjusted for each animal's day of sacrifice. NS: not significant after FDR (q ≥ 0.05). Significance: * p < 0.05; ** p < 0.01; *** p < 0.001. Bold entries indicate values that remain statistically significant after Benjamini–Hochberg FDR correction (q < 0.05; q_ANCOVA for the four ANCOVA comparisons and q for the Wilcoxon comparison). All measurements in cutaneous (thoracic skin) tissue homogenates. *Abbreviations: GPx, glutathione peroxidase; GSH, reduced glutathione; GSSG, oxidized glutathione; LPO/MDA, lipid peroxidation / malondialdehyde; PARP1, poly(ADP-ribose) polymerase 1; SOD, superoxide dismutase.*

Supplementary Table S9. Pulmonary biomarker responses to irradiation alone and in combination with olaparib.

Biomarker	Photons vs. control	Protons vs. control	Photons vs. Photons+Olaparib	Protons vs. Protons+Olaparib	Protons+Olaparib vs. Photons+Olaparib
**SOD**	NS	**-55.3%*** (d=2.27, p=<0.001, q=<0.001)**	NS	NS	NS
**Catalase**	-34.2%* (d=1.19, p=0.035, q=0.079)	**-48.8%** (d=1.69, p=0.005, q=0.009)**	NS	NS	NS
**GPx**	NS	NS	NS	NS	NS
**GSH**	**-69.6%*** (d=2.29, p=<0.001, q=<0.001)**	**-78.8%*** (d=2.53, p=<0.001, q=<0.001)**	NS	NS	NS
**GSSG**	+38.6%* (d=−0.94, p=0.046, q=0.083)	NS	NS	NS	NS
**GSH/GSSG**	**-77.8%*** (d=18.29, p=<0.001, q=<0.001)**	**-76.7%*** (d=14.51, p=<0.001, q=<0.001)**	NS	NS	NS
**LPO/MDA**	**-45.2%** (d=1.48, p=0.007, q=0.021)**	**-52.6%** (d=1.73, p=0.004, q=0.009)**	NS	+57.0%* (d=−1.19, p=0.029, q=0.261)	NS
**Protein**	NS	+34.7%* (d=−1.12, p=0.038, q=0.057)	NS	NS	NS
**Carbonylation**	NS	NS	NS	NS	NS

Note. Values are percentage changes relative to the indicated comparator group, with Cohen's d, p-value and Benjamini–Hochberg FDR q-value. The four time-shifted comparisons (Photons vs. control; Protons vs. control; Photons vs. Photons+Olaparib; Protons vs. Protons+Olaparib) were analysed by rank-based ANCOVA adjusted for the day of sacrifice, and report the day-adjusted p and its FDR-adjusted q (q_ANCOVA). The main comparison, protons + olaparib vs. photons + olaparib (median sacrifice day 8 after D0), was tested with a two-sided Wilcoxon–Mann–Whitney rank-sum test. P-values and FDR q-values are unadjusted for each animal's day of sacrifice. NS: not significant after FDR (q ≥ 0.05). Significance: * p < 0.05; ** p < 0.01; *** p < 0.001. Bold entries indicate values that remain statistically significant after Benjamini–Hochberg FDR correction (q < 0.05; q_ANCOVA for the four ANCOVA comparisons and q for the Wilcoxon comparison). All measurements in pulmonary tissue homogenates. *Abbreviations: GPx, glutathione peroxidase; GSH, reduced glutathione; GSSG, oxidized glutathione; LPO/MDA, lipid peroxidation / malondialdehyde; SOD, superoxide dismutase. PARP1 activity was not assessed in pulmonary tissue.*

Supplementary Table S10. Systemic biomarker responses to irradiation alone and in combination with olaparib.

Biomarker	Photons vs. control	Protons vs. control	Photons vs. Photons+Olaparib	Protons vs. Protons+Olaparib	Protons+Olaparib vs. Photons+Olaparib
Plasma — cytokines					
**IL-23**	NS	NS	−75.1%* (d=1.38, p=0.013, q=0.084)	NS	NS
**IL-1α**	NS	NS	NS	NS	NS
**IFN-γ**	+322.7%* (d=−1.08, p=0.038, q=0.078)	NS	NS	NS	NS
**TNF-α**	+158.6%* (d=−1.14, p=0.034, q=0.078)	NS	−60.3%* (d=1.13, p=0.039, q=0.169)	NS	NS
**MCP-1**	**+1785.5%** (d=−1.50, p=0.003, q=0.019)**	**+1669.3%** (d=−1.59, p=0.006, q=0.039)**	NS	NS	−69.5%* (d=−0.78, p=0.043, q=0.566)
**IL-12p70**	NS	NS	+∞%** (d=−1.50, p=0.008, q=0.084)	NS	NS
**IL-1β**	**+278.1%* (d=−1.41, p=0.012, q=0.045)**	NS	NS	NS	NS
**IL-10**	+128.9%* (d=−1.08, p=0.042, q=0.078)	NS	NS	NS	NS
**IL-6**	NS	**+1433.5%** (d=−1.63, p=0.005, q=0.039)**	NS	NS	NS
**IL-27**	**+559.2%* (d=−0.95, p=0.014, q=0.045)**	+522.6%* (d=−1.23, p=0.037, q=0.120)	NS	NS	NS
**IL-17A**	**+806.1%** (d=−1.33, p=0.001, q=0.013)**	+383.2%* (d=−1.47, p=0.012, q=0.052)	NS	NS	NS
**IFN-β**	NS	NS	NS	NS	NS
**GM-CSF**	NS	NS	NS	NS	NS
Plasma — other					
**Protein**	NS	NS	NS	NS	NS
**8-OXO-dG**	NS	NS	NS	NS	NS
**TGF-β**	**−89.4%*** (d=3.08, p=<0.001, q=<0.001)**	**−98.8%*** (d=3.45, p=<0.001, q=<0.001)**	NS	+521.2%* (d=−1.35, p=0.024, q=0.072)	NS
Erythrocytes					
**Protein**	NS	NS	**+63.6%* (d=−1.11, p=0.023, q=0.029)**	NS	NS

**Carbonylation**	**−31.7%** (d=1.56, p=0.002, q=0.014)**	**−49.0%*** (d=2.66, p=<0.001, q=<0.001)**	**−27.8%** (d=0.91, p=0.006, q=0.021)**	NS	NS
**Catalase**	NS	**−53.9%** (d=1.62, p=0.006, q=0.014)**	**−48.4%* (d=1.31, p=0.025, q=0.029)**	NS	NS
**GPx**	NS	NS	NS	NS	NS
**GSH**	**−37.9%* (d=1.42, p=0.011, q=0.038)**	**−51.4%** (d=1.83, p=0.003, q=0.011)**	**−25.2%* (d=1.43, p=0.012, q=0.021)**	+49.8%* (d=−1.42, p=0.015, q=0.052)	**+56.9%** (d=1.76, p=0.008, q=0.027)**
**GSSG**	NS	**−39.0%* (d=1.41, p=0.013, q=0.023)**	**−24.8%* (d=1.44, p=0.012, q=0.021)**	+54.6%** (d=−1.55, p=0.009, q=0.052)	**+53.3%** (d=1.75, p=0.003, q=0.021)**
**SOD**	NS	NS	**−27.1%** (d=1.66, p=0.004, q=0.021)**	NS	**+80.8%* (d=1.38, p=0.017, q=0.04)**
Lymphocytes					
**Protein**	−36.1%* (d=1.46, p=0.047, q=0.078)	NS	NS	NS	NS
**8-OXO-dG**	+75.2%* (d=−1.50, p=0.035, q=0.078)	NS	NS	NS	NS
**Carbonylation**	NS	**−79.8%*** (d=5.27, p=<0.001, q=<0.001)**	NS	NS	NS
**LPO/MDA**	NS	NS	NS	NS	NS
**PARP1 activity**	+71.5%* (d=−1.16, p=0.010, q=0.050)	NS	−31.4%* (d=1.01, p=0.038, q=0.171)	NS	NS

Note. Values are percentage changes relative to the indicated comparator group, with Cohen's d, p-value and Benjamini–Hochberg FDR q-value. The four time-shifted comparisons (Photons vs. control; Protons vs. control; Photons vs. Photons+Olaparib; Protons vs. Protons+Olaparib) were analysed by rank-based ANCOVA adjusted for the day of sacrifice (day-adjusted p and FDR q_ANCOVA). The main comparison, protons + olaparib vs. photons + olaparib (median sacrifice day 8 after D0), was tested with a two-sided Wilcoxon–Mann–Whitney rank-sum test. P-values and FDR q-values are unadjusted for each animal's day of sacrifice. Cytokines, TGF-β1 and 8-OXO-dG were measured in plasma; erythrocyte and lymphocyte markers in the corresponding blood cell fractions. NS: not significant (p ≥ 0.05); +∞% denotes an increase from an undetectable (zero) comparator value. Significance: * p ≤ 0.05; ** p < 0.01; *** p < 0.001. Bold entries indicate values that remain statistically significant after Benjamini–Hochberg FDR correction (q < 0.05; q_ANCOVA for the four ANCOVA comparisons and q for the Wilcoxon comparison). *Abbreviations: GM-CSF, granulocyte–macrophage colony-stimulating factor; GPx, glutathione peroxidase; GSH, reduced glutathione; GSSG, oxidized glutathione; IFN, interferon; IL, interleukin; LPO/MDA, lipid peroxidation / malondialdehyde; MCP-1, monocyte chemoattractant protein-1; 8-OXO-dG, 8-oxo-2'-deoxyguanosine; PARP1, poly(ADP-ribose) polymerase 1; SOD, superoxide dismutase; TGF-β, transforming growth factor-β; TNF-α, tumor necrosis factor-α.*

Supplementary Table S11. Rank-based ANCOVA of the biomarkers adjusted for the day of sacrifice, across all treatment comparisons and tissue compartments. Only biomarkers remaining significant after adjustment and FDR correction (q_ANCOVA < 0.05) are shown, ordered by tissue as cited in the main text.

Biomarker	Comparison	pvalue	d	delta	p_ANCOVA	q Unadjusted Wilcoxon	q Day-adjusted
**Cerebral**							
Catalase	Photons vs. control	0.023*	-1.59	+103.7%	0.003**	0.076	**0.015**
GSH/GSSG	Photons vs. control	0.011*	1.61	−52.2%	0.009**	0.058	**0.030**
GSSG	Photons vs. control	0.005**	-2.03	+186.6%	0.001**	0.052	**0.010**
GSH/GSSG	Protons vs. control	0.005**	1.76	−56.2%	0.005**	0.017	**0.017**
LPO	Protons vs. control	0.001**	2.49	−49.2%	<0.001***	0.006	**<0.001**
SOD	Protons vs. control	<0.001***	3.57	−50.7%	<0.001***	0.002	**<0.001**
Catalase	Photons vs. Photons+Ola	0.026*	1.54	−49.0%	0.011*	0.058	**0.037**
GPx	Photons vs. Photons+Ola	<0.001***	2.10	−79.5%	0.002**	0.003	**0.010**
PARP	Photons vs. Photons+Ola	0.001**	1.90	−65.1%	0.002**	0.006	**0.010**
**Cardiac**							
GSH	Photons vs. control	<0.001***	1.77	−89.8%	0.003**	<0.001	**0.010**
GSH/GSSG	Photons vs. control	<0.001***	-4.12	+33.5%	<0.001***	<0.001	**<0.001**
GSSG	Photons vs. control	<0.001***	1.86	−92.2%	0.002**	<0.001	**0.010**
GSH/GSSG	Protons vs. control	<0.001***	3.73	−18.2%	<0.001***	0.002	**<0.001**
GSH/GSSG	Photons vs. Photons+Ola	0.028*	1.83	−26.1%	0.002**	0.040	**0.010**
Protein	Photons vs. Photons+Ola	<0.001***	2.14	−50.6%	0.001**	0.002	**0.010**
**Hepatic**							
Carbonylation†	Photons vs. control	0.018*	-1.47	+138.9%	0.001**	0.046	**0.005**
GSH†	Photons vs. control	0.027*	-1.14	+66.3%	0.024*	0.053	**0.048**
GSSG†	Photons vs. control	<0.001***	-1.60	+118.1%	0.001**	0.005	**0.005**
PARP†	Photons vs. control	0.015*	-1.18	+165.5%	0.020*	0.046	**0.048**
Protein	Photons vs. control	0.015*	1.49	−32.2%	0.002**	0.046	**0.007**
Carbonylation†	Protons vs. control	0.013*	-1.53	+270.2%	0.017*	0.014	**0.024**
GPx†	Protons vs. control	<0.001***	1.75	−81.7%	0.006**	<0.001	**0.010**
GSH†	Protons vs. control	<0.001***	2.14	−73.5%	0.001**	<0.001	**0.003**
GSSG†	Protons vs. control	<0.001***	2.60	−72.2%	<0.001***	<0.001	**<0.001**
LPO†	Protons vs. control	<0.001***	2.74	−60.4%	<0.001***	<0.001	**<0.001**
PARP†	Protons vs. control	0.011*	1.35	−41.2%	0.025*	0.014	**0.031**
Protein	Protons vs. control	0.001**	-4.12	+176.6%	<0.001***	0.002	**<0.001**
SOD†	Protons vs. control	0.003**	2.03	−59.8%	0.002**	0.004	**0.004**
GSH/GSSG	Protons vs. Protons+Ola	0.005**	-1.91	+55.2%	0.003**	0.048	**0.030**
GSSG†	Protons vs. Protons+Ola	0.018*	1.69	−63.1%	0.009**	0.059	**0.045**
**Intestinal**							
Catalase	Protons vs. control	<0.001***	-2.84	+44.0%	<0.001***	0.007	**<0.001**
SOD	Protons vs. control	0.003**	-1.55	+166.7%	0.004**	0.013	**0.020**
GSH/GSSG	Protons vs. Protons+Ola	<0.001***	-4.87	+58.3%	<0.001***	0.002	**<0.001**
GSSG	Protons vs. Protons+Ola	<0.001***	2.81	−45.1%	<0.001***	0.002	**<0.001**
**Cutaneous**							
Catalase	Photons vs. control	0.009**	1.56	−45.8%	0.005**	0.028	**0.025**
GSSG	Photons vs. control	0.009**	1.48	−59.8%	0.008**	0.028	**0.027**
PARP	Photons vs. control	0.004**	-1.67	+161.2%	0.004**	0.028	**0.025**
LPO	Protons vs. control	<0.001***	1.97	−68.5%	0.002**	0.003	**0.016**
**Pulmonary**							
GSH	Photons vs. control	<0.001***	2.29	−69.6%	<0.001***	<0.001	**<0.001**
GSH/GSSG	Photons vs. control	<0.001***	18.29	−77.8%	<0.001***	<0.001	**<0.001**
LPO	Photons vs. control	0.003**	1.48	−45.2%	0.007**	0.009	**0.021**
Catalase	Protons vs. control	0.005**	1.69	−48.8%	0.005**	0.008	**0.009**
GSH	Protons vs. control	<0.001***	2.53	−78.8%	<0.001***	<0.001	**<0.001**
GSH/GSSG	Protons vs. control	<0.001***	14.51	−76.7%	<0.001***	<0.001	**<0.001**
LPO	Protons vs. control	<0.001***	1.73	−52.6%	0.004**	0.002	**0.009**
SOD	Protons vs. control	<0.001***	2.27	−55.3%	<0.001***	0.002	**<0.001**
**Systemic/blood**							
Carbonylation (erythrocytes)	Photons vs. control	0.009**	1.56	−31.7%	0.002**	0.030	**0.014**
GSH (erythrocytes)	Photons vs. control	0.002**	1.42	−37.9%	0.011*	0.015	**0.038**
IL-17A (plasma)	Photons vs. control	0.006**	-1.33	+806.1%	0.001**	0.025	**0.013**
IL-1β (plasma)	Photons vs. control	0.043*	-1.41	+278.1%	0.012*	0.073	**0.045**

IL-27 (plasma)	Photons vs. control	0.045*	-0.95	+559.2%	0.014*	0.073	**0.045**
MCP-1 (plasma)	Photons vs. control	0.001**	-1.50	+1785.5%	0.003**	0.008	**0.019**
TGF-β1 (plasma)	Photons vs. control	<0.001***	3.08	−89.4%	<0.001***	0.002	**<0.001**
Carbonylation (erythrocytes)	Protons vs. control	0.002**	2.66	−49.0%	<0.001***	0.005	**<0.001**
Carbonylation (lymphocytes)	Protons vs. control	0.006**	5.27	−79.8%	<0.001***	0.030	**<0.001**
Catalase (erythrocytes)	Protons vs. control	0.003**	1.62	−53.9%	0.006**	0.007	**0.014**
GSH (erythrocytes)	Protons vs. control	<0.001***	1.83	−51.4%	0.003**	0.005	**0.011**
GSSG (erythrocytes)	Protons vs. control	0.010**	1.41	−39.0%	0.013*	0.017	**0.023**
IL-6 (plasma)	Protons vs. control	0.013*	-1.63	+1433.5%	0.005**	0.042	**0.039**
MCP-1 (plasma)	Protons vs. control	<0.001***	-1.59	+1669.3%	0.006**	0.001	**0.039**
TGF-β1 (plasma)	Protons vs. control	<0.001***	3.45	−98.8%	<0.001***	<0.001	**<0.001**
Carbonylation (erythrocytes)	Photons vs. Photons+Ola	0.114	0.91	−27.8%	0.006**	0.133	**0.021**
Catalase (erythrocytes)	Photons vs. Photons+Ola	0.038*	1.31	−48.4%	0.025*	0.066	**0.029**
GSH (erythrocytes)	Photons vs. Photons+Ola	0.015*	1.43	−25.2%	0.012*	0.035	**0.021**
GSSG (erythrocytes)	Photons vs. Photons+Ola	0.008**	1.44	−24.8%	0.012*	0.028	**0.021**
Protein (erythrocytes)	Photons vs. Photons+Ola	0.114	-1.11	+63.6%	0.023*	0.133	**0.029**
SOD (erythrocytes)	Photons vs. Photons+Ola	0.008**	1.66	−27.1%	0.004**	0.028	**0.021**

Note. Each row is a biomarker×comparison combination, grouped by tissue compartment in the order the compartments are discussed in the Results. Only combinations remaining significant after rank-based ANCOVA adjusting for the day of sacrifice and Benjamini–Hochberg FDR correction (q_ANCOVA < 0.05, shown in bold) are listed. Columns: pvalue, unadjusted Wilcoxon rank-sum p-value; d, Cohen's d effect size; delta, percentage change relative to the comparator; p_ANCOVA, day-adjusted ANCOVA p-value; q, Benjamini–Hochberg FDR value of the unadjusted p; q_ANCOVA, Benjamini–Hochberg FDR value of the day-adjusted p. Only the four time-shifted comparisons analysed by ANCOVA are shown; the two contrasts analysed without day adjustment (control vs. olaparib; protons+olaparib vs. photons+olaparib) are reported separately. Significance: * $p \leq 0.05$; ** $p < 0.01$; *** $p < 0.001$; +∞ denotes an increase from an undetectable (zero) comparator value. † Per mg of total hepatic protein, which differed markedly between groups ($q < 0.001$); not interpretable, shown for transparency. *Abbreviations: GPx, glutathione peroxidase; GSH, reduced glutathione; GSSG, oxidized glutathione; IL, interleukin; LPO, lipid peroxidation; MCP-1, monocyte chemoattractant protein-1; PARP, poly(ADP-ribose) polymerase; SOD, superoxide dismutase; TGF-β1, transforming growth factor-β1.*

Supplementary Figure S5. Standardized effect sizes for biomarkers differing between the two combination arms.

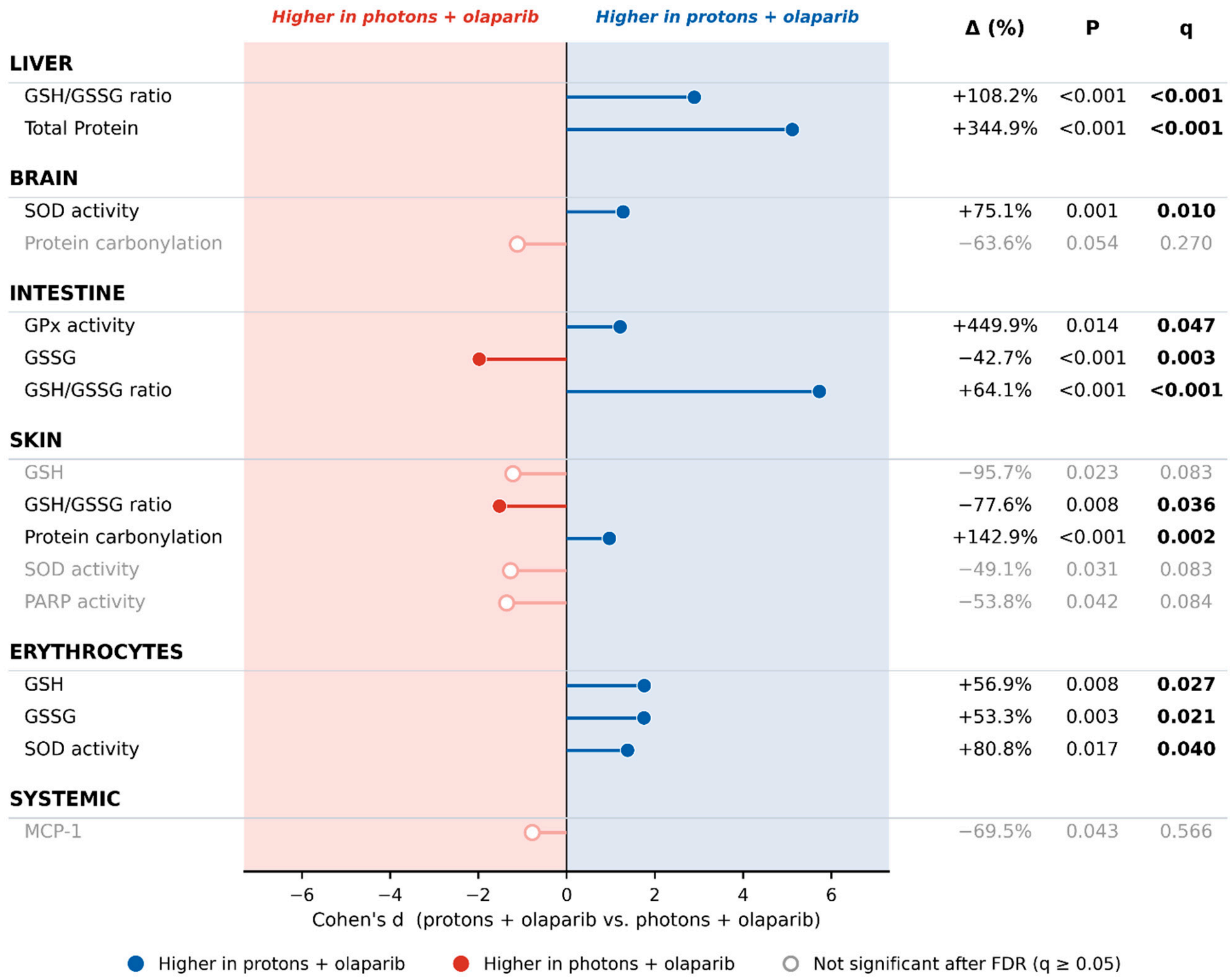

Note: Standardized effect sizes (Cohen's d) for the interpretable biomarkers showing a nominal between-arm difference in the principal comparison of this exploratory study (protons + olaparib vs. photons + olaparib; two-sided Wilcoxon–Mann–Whitney test), grouped by tissue. Filled circles denote biomarkers surviving Benjamini–Hochberg FDR correction ($q < 0.05$; $n = 11$); open circles denote nominal differences not surviving FDR correction ($n = 5$). Blue, higher in protons + olaparib; red, higher in photons + olaparib. Δ (%), p and q columns report the percentage change, nominal p-value and FDR-corrected q-value.
